# Growth and stress response mechanisms underlying post-feeding regenerative organ growth in the Burmese python

**DOI:** 10.1186/s12864-017-3743-1

**Published:** 2017-05-02

**Authors:** Audra L. Andrew, Blair W. Perry, Daren C. Card, Drew R. Schield, Robert P. Ruggiero, Suzanne E. McGaugh, Amit Choudhary, Stephen M. Secor, Todd A. Castoe

**Affiliations:** 1 Department of Biology, The University of Texas Arlington, 501 S. Nedderman Dr, Arlington, TX 76019 USA; 2 Department of Biology, New York University Abu Dhabi, Saadiyat Island, Abu Dhabi, United Arab Emirates; 3 Department of Ecology, Evolution, and Behavior, University of Minnesota, St. Paul, MN 55108 USA; 4 Harvard Medical School, Renal Division, Brigham and Woman’s Hospital, Cambridge, MA 02142 USA; 5 Center for the Science of Therapeutics, Broad Institute, Cambridge, MA 02142 USA; 6 Department of Biological Sciences, University of Alabama, Tuscaloosa, AL 35487 Box 870344, USA

**Keywords:** Hyperplasia, Hypertrophy, NRF2, mTOR, Regeneration, RNAseq

## Abstract

**Background:**

Previous studies examining post-feeding organ regeneration in the Burmese python (*Python molurus bivittatus*) have identified thousands of genes that are significantly differentially regulated during this process. However, substantial gaps remain in our understanding of coherent mechanisms and specific growth pathways that underlie these rapid and extensive shifts in organ form and function. Here we addressed these gaps by comparing gene expression in the Burmese python heart, liver, kidney, and small intestine across pre- and post-feeding time points (fasted, one day post-feeding, and four days post-feeding), and by conducting detailed analyses of molecular pathways and predictions of upstream regulatory molecules across these organ systems.

**Results:**

Identified enriched canonical pathways and upstream regulators indicate that while downstream transcriptional responses are fairly tissue specific, a suite of core pathways and upstream regulator molecules are shared among responsive tissues. Pathways such as mTOR signaling, PPAR/LXR/RXR signaling, and NRF2-mediated oxidative stress response are significantly differentially regulated in multiple tissues, indicative of cell growth and proliferation along with coordinated cell-protective stress responses. Upstream regulatory molecule analyses identify multiple growth factors, kinase receptors, and transmembrane receptors, both within individual organs and across separate tissues. Downstream transcription factors MYC and SREBF are induced in all tissues.

**Conclusions:**

These results suggest that largely divergent patterns of post-feeding gene regulation across tissues are mediated by a core set of higher-level signaling molecules. Consistent enrichment of the NRF2-mediated oxidative stress response indicates this pathway may be particularly important in mediating cellular stress during such extreme regenerative growth.

**Electronic supplementary material:**

The online version of this article (doi:10.1186/s12864-017-3743-1) contains supplementary material, which is available to authorized users.

## Background

The ability to massively downregulate metabolic and physiological functions during extended periods of fasting has evolved in multiple species of snakes. This downregulation of physiological form includes the atrophy of organs such as the heart, kidney, liver, and small intestine. Upon feeding, the size and function of these organs, along with oxidative metabolism, is massively upregulated to accommodate digestion [1–4]. Of the snake species that experience these large fluctuations in physiology, the Burmese python (*Python molurus bivittatus*) is the most well-studied [5]. Within 48 h of feeding, Burmese pythons can undergo up to a 44-fold increase in metabolic rate and >100-fold increases in plasma triglyceride content [3, 6]. Major organs also experience dramatic shifts in physiological form, including 40–100% increases in the mass of the heart, liver, pancreas, kidneys, and small intestine [2, 7–10]. This extreme organ regenerative growth and atrophy is unparalleled across vertebrates, and studies indicate that this organ growth is driven by multiple cellular processes, including cellular hypertrophy in the heart and mixtures of hypertrophy and hyperplasia in the kidney, liver, and small intestine [3, 5, 11, 12]. Organ growth peaks around 1–2 days post-feeding (DPF), and by 10–14DPF, organ form and function, as well as gene expression patterns, have completely reversed back to fasted levels [1, 2, 5, 7–9, 13].

Previous studies have examined aspects of this post-feeding response using morphological and physiological assays [2, 3, 7, 14–16], analyses of gene expression [5, 13], and combinations of the two [5, 14]. Together, these studies have demonstrated that transcriptional responses following feeding are extremely rapid and massive, both in the magnitude of expression changes and in the number of genes with significant differential expression. Genes important in a number of developmental, metabolic, proliferative, apoptotic, and growth processes have been shown to be involved in these major shifts in organ form and function [5, 11, 13]. Previous studies have shown that mammalian cells respond to the growth signals in post-fed python serum, which likely indicates a conserved response to core signaling molecules [11, 17]. We therefore hypothesize that a relatively small number of core molecular regulatory molecules and signaling pathways may underlie these responses. However, the identification of a core set of upstream regulatory molecules and mechanisms has been hindered by the large number of genes that are significantly differentially expressed during this response, making manual interpretation of this gene expression data difficult. Additionally, the lack of comparable replicated sampling across multiple organs has further prevented meaningful across-organ comparisons of changes in gene expression in previous studies [13]. Accordingly, major gaps remain in our understanding of the specific mechanisms and growth pathways that are responsible for driving these extreme shifts in Burmese python organ size and function, as well as how these mechanisms may vary across different organ systems.

Our previous study of the Burmese python feeding response addressed some gaps through the use of increased replicates and more frequent time point sampling for one organ, the small intestine [5]. We identified over 1,700 genes that were significantly differentially expressed during post-feeding regeneration in the small intestine with many of these genes being functionally linked to cellular processes such as WNT signaling, cell cycling, and apoptosis. This study also linked changes in gene expression with functional and phenotypic shifts by comparing RNAseq data with physiological and histological data. This detailed analysis was only conducted on the small intestine, however, and failed to address any upper-level signaling mechanisms and pathways.

Here, we leverage fully replicated organ-specific time courses detailing gene-level responses to infer canonical pathways and regulatory molecules driving post-feeding organ growth in the Burmese python. We examined gene expression across four major organ systems – the heart, liver, kidney, and small intestine. We combined increased replicated sampling with statistical inferences of pathway activation and regulatory molecule prediction to identify the mechanistic drivers of cross-tissue, post-feeding organ regeneration. Despite highly organ-specific gene expression responses associated with organ regenerative growth, we found evidence for high degrees of overlap in predicted pathways and regulatory molecules underlying these growth processes between organs. Pathways predicted to be involved in regulating this physiological response include LXR/RXR activation, PI3K/AKT, and mTOR signaling. Interestingly, we also found strong and consistent evidence for the involvement of NRF2-mediated oxidative stress response and other stress-response pathways in this extreme example of rapid organ growth. Our results suggest that post-feeding, regenerative organ growth in the Burmese python may stem from small numbers of key effector molecules mediating a core set of growth and stress-response pathways, which in turn activate diverse, tissue-specific signaling cascades.

## Methods

### Feeding experiments

Burmese pythons were obtained from commercial breeders. All animal care and tissue sampling was conducted using protocols approved by the University of Alabama Institutional Animal Care and Use Committee (14-06-0075). Burmese pythons were sampled at three physiological states: fasted (30 days since last meal), 1 day post-feeding (1DPF) and 4DPF, with the meal consumed equaling at least 25% of their body mass. Previous studies have shown that organ masses and functional phenotypes climax between 1 and 3 DPF [1, 2, 5, 9] and that phenotypes begin to decline by 4DPF [2, 3, 7, 9]. We therefore chose sampling time points here to capture gene expression patters during the period before phenotypes climax (1DPF) and early in their regression (4DPF). Snakes were humanely euthanized by severing the spinal cord immediately behind the head, and organs were immediately extracted, snap frozen in liquid nitrogen, and stored at -80 °C. Between three and six biological replicates (i.e., animals) were sampled for each time point. See Additional file 1, Supplementary Methods for additional details.

### Transcriptome library generation

Total RNA was extracted from ~50 mg of snap-frozen tissue using Trizol Reagent (Invitrogen), followed by mechanical cell disruption using a TissueLyzer for 10 min at 20 strokes/min, and precipitation of RNA using isopropanol. Individual Illumina mRNAseq libraries were constructed using either the Illumina TruSeq RNAseq kit or the NEB Next RNAseq kit, both of which included poly-A selection, RNA fragmentation, cDNA synthesis, and indexed Illumina adapter ligation. Completed RNAseq libraries were quantified on a BioAnalyzer (Agilent), pooled in equal molar ratios in various multiplex arrangements, and sequenced on either an Illumina GAIIx or Illumina HiSeq2000 (see Additional file 1: Table S1).

### Quantifying and visualizing gene expression

Raw demultiplexed Illumina RNAseq reads were quality filtered and trimmed with Trimmomatic v. 0.32 [18]. In instances where the same library was sequenced in multiple different runs, reads were combined and mapped for each individual and time point. Mapping of reads to the reference transcriptome of the Burmese python [13] was conducted using BWA v. 0.6.1 [19] with the following parameters: mismatch penalty = 2, gap open penalty = 3, and alignment score minimum = 20. Expression was determined using SAMtools v. 0.1.19 [20] by counting the number of unique gene reads that mapped to an annotated transcript, while excluding reads that mapped to multiple positions. New RNAseq data for various time points and replicates was analyzed together with previously published data from other individuals and replicates [5, 13]. Newly-generated sequencing data were archived on the NCBI Short Read Archive (NCBI: SRP051827).

Raw expression counts were normalized using TMM normalization in edgeR [21] and all statistical analyses of gene expression were conducted using normalized data. We identified genes that were significantly differentially expressed between time points using two approaches. First, we estimated significant changes in gene expression between pairs of time points using pairwise exact tests for the binomial distribution calculated in edgeR, integrating both common and tagwise dispersion [21]. Second, to accommodate the time-series nature of the experimental design, we also conducted step-wise regression analysis of gene expression in maSigPro [22]. Regression analysis enabled the detection of genes with significant patterns of differential expression across all three time points. Gene expression heatmaps were generated in R and clustered with the package vegan [23], with gene clustering calculated using average linkage hierarchical clustering based on a Bray-Curtis dissimilarity matrix. We used the program STEM [24] to identify and visualize significant expression profiles for all genes in our RNAseq data.

### Assigning homology for functional analyses

To facilitate the use of various pathway activation and regulatory molecule predictions, we annotated the full Burmese python transcript set [13] with orthologous human gene Ensembl [25] identifiers. Reciprocal tblastx was first conducted between *Anolis carolinensis* and Burmese python, and *Anolis* gene IDs identified as orthologous to python genes were converted to human Ensembl identifiers using homology tables from Ensembl’s Biomart [26]. The same process of reciprocal best blast using tblastx was performed between Burmese python and *Gallus gallus*, followed by conversion of chicken Ensembl identifiers to human Ensembl identifiers using homology tables from Ensembl’s Biomart [26]. We also performed reciprocal best blast of the python with *Homo sapiens*. Finally, we used one-way tblastx with anolis, chicken, and human to annotate python genes that were not assigned an ortholog from reciprocal best blast. Using this annotation approach, we were able to assign human Ensembl IDs to 22,393 of 25,385 total python reference transcripts.

### Pathway and upstream regulatory molecule analyses

**Fig. 1:**
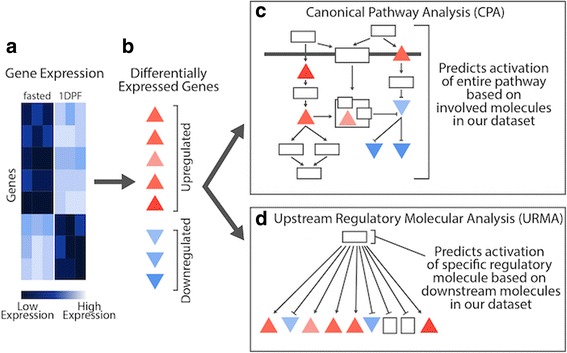
Conceptual overview of differences between Canonical Pathway Analysis (CPA) and Upstream Regulatory Molecule Analysis (URMA). Pairwise analyses on experimental gene expression data (**a**) identify significantly upregulated and downregulated genes (**b**). Significantly differentially expressed genes are then analyzed in two distinct IPA analyses (CPA and URMA) (**c**) Canonical Pathway Analysis predicts pathway activation based on overlap of gene expression data with molecules within the pathway. **d** Upstream Regulatory Molecule Analysis predicts activation of specific regulatory molecules based on downstream molecules in our gene expression dataset

To infer the involvement of upstream regulatory molecules and pathways, we performed Core Analysis in Ingenuity Pathway Analysis (IPA; Qiagen), using default parameters. IPA uses gene identifiers and the fold-change value for each differentially expressed gene to identify enrichment patterns for Canonical Pathway Analysis (CPA) and Upstream Regulatory Molecule Analysis (URMA), and to infer the activation direction (activated versus inhibited) between particular time points. These two analyses both use observed gene expression data to infer unobserved features (e.g., activation state of key signaling molecules), but differ fundamentally in how they use expression data to make inferences. CPA predicts the involvement and activation/inhibition of canonical pathways based on observed evidence from gene expression data, specifically for genes that participate as higher-level regulatory molecules within a given pathway; analysis of observed gene expression data incorporates information from the Ingenuity Knowledge Base (including genes known to be involved within a given pathway) to provide both a statistical value of enrichment and a prediction of the biological involvement for the pathway as a whole (i.e. activated or inhibited; IPA documentation, Qiagen). In contrast, URMA uses observed changes in gene expression specifically for genes at lower levels within pathways (e.g., low level effectors) to predict activation or inhibition of regulatory molecules upstream of these genes [27]. Due to differences in these approaches, together these two methods provide a well-rounded set of comparable inferences for dissecting molecular mechanisms (Fig. 1).

For IPA analyses, we used only genes identified as significant in pairwise differential expression analyses between time intervals (per organ), and we input fold changes per gene averaged across biological replicates, along with our estimate of the orthologous human Ensembl ID for each gene. Pathways important to cross-tissue physiological responses were isolated using the IPA CPA (included with Core Analysis), with a right-tailed Fisher’s exact test *p*-value of less than 0.01. We examined only those pathways that were significant, based on a predicted activation z-score, in at least one of the four organs for at least one of the post-feeding time points. For IPA analyses, the z-score is used to determine the statistical significance of the number of activated and inhibited predictions, and the sign of the value indicates the overall activation state (i.e., positive versus negative activation). We used a *p*-value cutoff of 0.01 for the CPA in IPA to reduce potentially spurious inferences. Upstream regulators and hypotheses for global signaling molecules were identified using URMA in IPA, with a Fisher’s exact test overlap *p*-value threshold of 0.05. Pathway network figures were modified manually from predicted network figures generated in IPA. For analysis of specific pathways (mTOR signaling and NRF2-mediated oxidative stress response), we also determined the number of genes involved in each pathway that were assigned python orthologs by our orthology analyses, and how many of these genes were expressed at some level in our dataset (see Additional file 1: Table S2).

## Results

### Trends in gene expression across organs

We used our expression data from all python samples (see Additional file 1: Table S1) to examine the degree to which different organ systems ‘turn on’ upon feeding and then experience ‘regression’ towards pre-feeding patterns of expression at 4DPF. We found that for each organ, the majority of differentially expressed genes showed immediate up- or downregulation from fasting to 1DPF. Interestingly, each of the four organs examined appeared to experience regression towards fasting levels of expression by 4DPF to widely different extents, indicating that each organ may have its own unique temporal program of growth followed by atrophy. Across organs, the heart appeared to shift towards regression the fastest. Other organs experienced reversals of fasted to 1DPF expression shifts to varying degrees by 4DPF, ranging from the moderately paced small intestine and kidney, to the slow-paced liver (Table 1). STEM analysis further supported these temporal patterns of up-regulation and regression across organs (see Additional file 1: Figure S1).

**Table 1:** Numbers of differentially expressed genes between pre- and post-feeding time points for the four organs studied

	Time point Comparisons					
	Fasted v 1DPF		1DPF v 4DPF		Fasted v 4DPF	
	Up	Down	Up	Down	Up	Down
Heart	208	228	36	40	5	3
Kidney	244	100	5	3	125	22
Liver	335	126	29	12	295	76
Small Intestine	1,271	1,042	268	146	547	345

For each comparison, the numbers of up and downregulated genes were inferred using pairwise analysis with a Benjamini-Hochberg corrected *p*-value <0.05

**Fig. 2:**
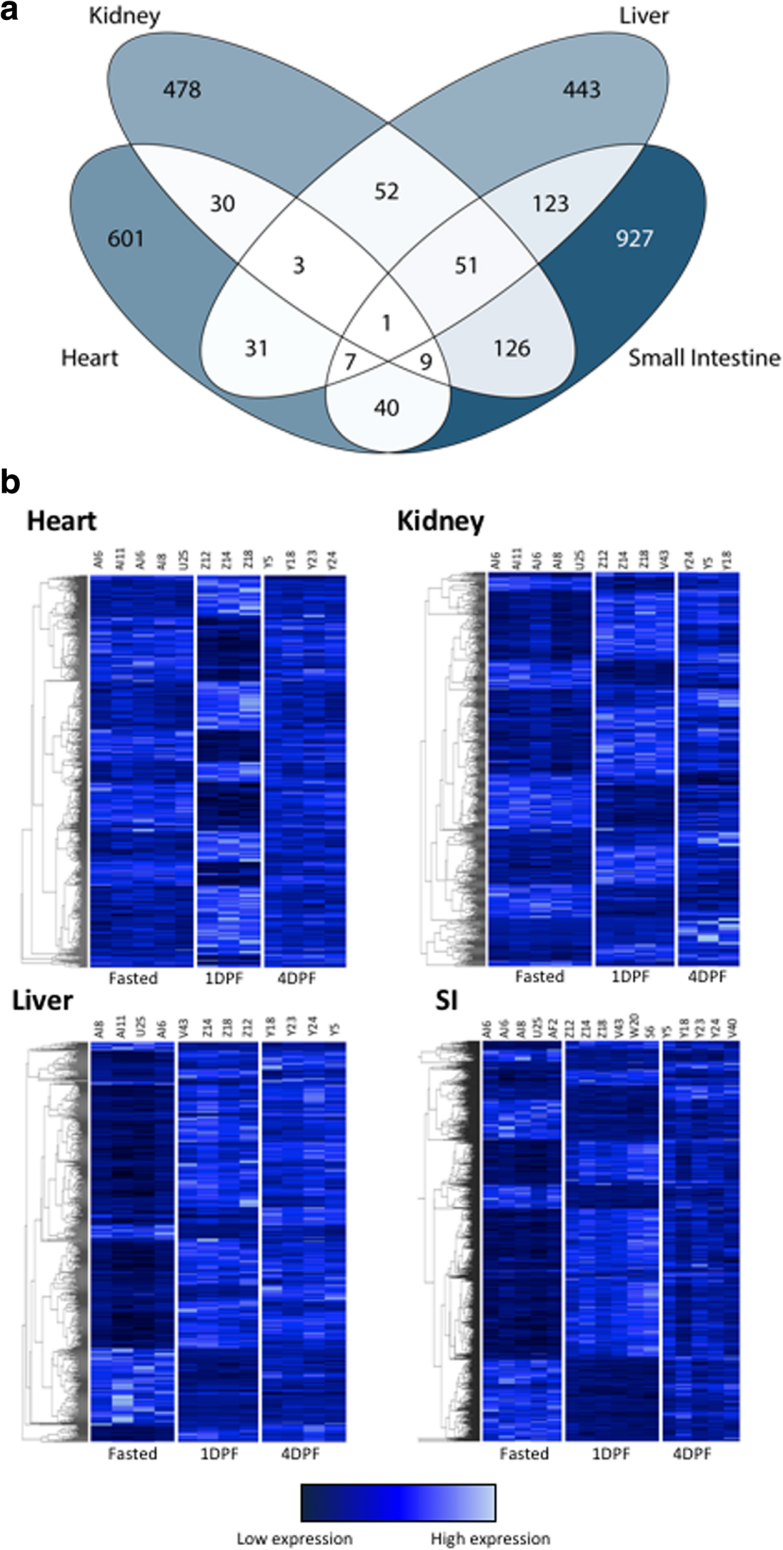
Summary of significantly differentially expressed genes for all four organs identified via regression analysis. **a** Venn diagram depicting the numbers of genes significantly differentially expressed across time points. Darker colors indicate a large number of genes and lighter colors indicate a smaller number of genes. **b** Heatmaps depicting all significantly differentially expressed genes across all time points in each organ. 722 genes were significantly differentially expressed in the heart. There were 750 genes significantly differentially expressed in the kidney. 711 genes were significantly differentially expressed in the liver and 1,284 genes showed significant differential expression in the small intestine

Regression analysis across time points, which tends to be conservative, identified hundreds of genes that were significantly differentially expressed across all three time points with 722 genes in the heart, 750 genes in the kidney, 711 genes in the liver, and 1,284 genes in the small intestine. Of the 2,922 total genes differentially expressed across all four organs, 21% are unique to the heart, 16% are unique to the kidney, 15% are unique to the liver, and 32% are unique to the small intestine (Fig. 2). Only a single gene was identified as significant in all four organs across all time points: *coagulation factor X* (*F10*).

To further dissect patterns of expression change following feeding, we conducted pairwise analyses of gene expression between time points for each organ. In the heart, pairwise analyses identified 436 significantly differentially expressed genes between fasted and 1DPF (208 upregulated and 228 downregulated; Table 1), and 76 genes were significantly differentially expressed between 1DPF and 4DPF (36 upregulated and 40 downregulated). In the kidney, 344 genes were significantly differentially expressed between the fasted state and 1DPF (244 upregulated and 100 downregulated), while only eight genes were significantly differentially expressed from 1DPF to 4DPF (five upregulated and three downregulated). In contrast to the heart, we found many genes (147) significantly differentially expressed between fasted and 4DPF in the kidney. In the liver, 461 genes were differentially expressed within 1DPF (335 upregulated and 126 downregulated), while only 41 genes were significantly differentially expressed from 1DPF to 4DPF (29 upregulated and 12 downregulated). With 371 genes significantly differentially expressed between fasted and 4DPF, among all four organs, the liver was the least ‘reset’ to the fasting condition by 4DPF. Finally, the small intestine showed higher levels of differential expression than the other three organs. Within 1DPF, 2,313 genes were significantly differentially expressed (1,271 upregulated and 1,042 downregulated). From 1DPF to 4DPF, 268 genes were upregulated and 146 genes were downregulated, and 892 genes were differentially expressed between fasted and 4DPF (Table 1).

### Genes and pathways implicated in differential gene expression in individual tissues

**Fig. 3:**
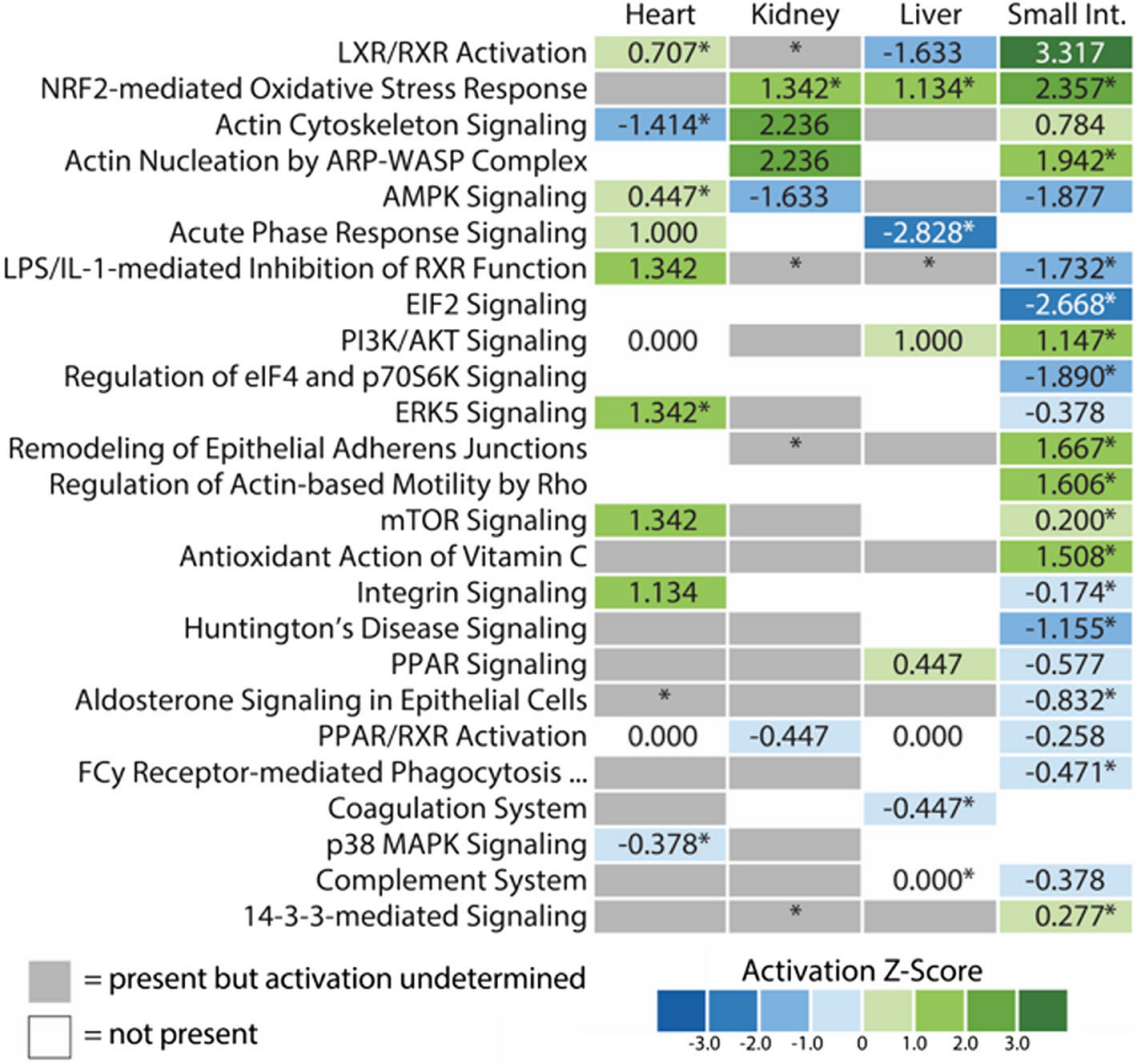
Canonical pathways predicted to be activated or inhibited from gene expression data. Each pathway shown is significantly enriched for our genes with a Fisher’s Exact test *p*-value less than 0.01 (depicted with an asterisk). Pathways were shown only if they met our criteria for significance and had a predicted activation state in at least one organ. Z-scores of 0.000 indicate pathway predictions that lack a bias in the direction of gene regulation observed in our dataset. PPAR signaling (*P* < 0.05) was also included

To move beyond gene-specific responses and towards deciphering the mechanisms that may underlie growth responses across different organs, we identified pathways that were significantly activated/repressed between fasting and 1DPF (Fig. 3). We found consistent evidence that the NRF2 stress-response pathway is activated in all tissues, except in the heart, where there was insufficient data to determine the direction of activation. We also found relatively consistent evidence for activation of the related growth pathways mTOR and PI3K/AKT across organs, although this inference was most significant in the heart and small intestine. We also inferred the involvement of the related pathways: LXR/RXR, LPS/IL-1-mediated inhibition of RXR function, PPAR/RXR, and PPAR signaling in multiple organs; the direction of stimulation of these pathways was both variable across organs and inconclusive in some organs. Substantial involvement of cytoskeletal pathways, including Actin cytoskeleton signaling and Actin nucleation by ARP-WASP complex, was also inferred across organs and positive in the kidney and small intestine, yet negative or inconclusive for the heart and liver, respectively.

In addition to pathway activation/repression patterns shared across organs, a number of pathways showed substantial organ-specific directionality of response. Examples of this pattern include the growth-related AMPK signaling pathway (which was activated in the heart, repressed in the kidney and small intestine, and ambiguous in the liver), ERK5 signaling (activated in the heart and repressed in the small intestine), and Integrin signaling (stimulated in the heart and repressed in the small intestine). Lastly, a number of pathways appeared to be organ-specific, including p38 MAPK and ERK5 signaling in the heart and 14-3-3-mediated signaling in the small intestine (Fig. 3).

### Upstream regulatory molecule analysis of 1 DPF responses

**Fig. 4:**
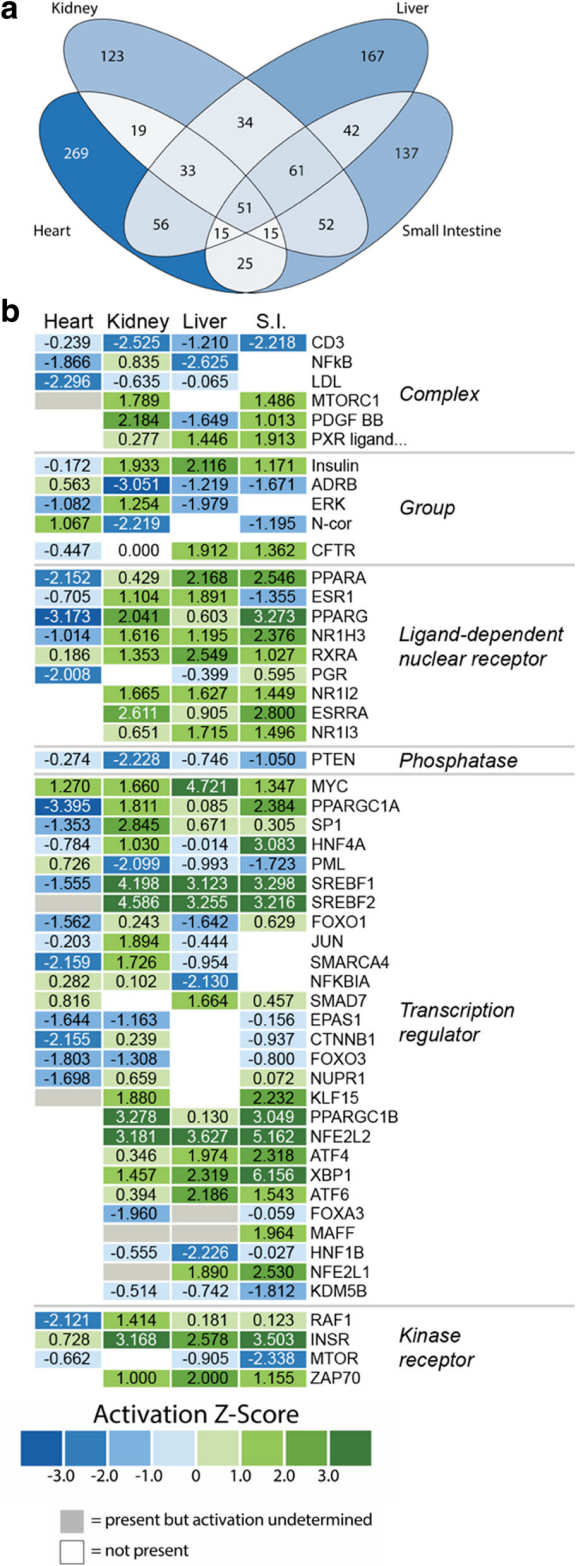
Predicted upstream regulators from IPA analysis of gene expression changes from fasted to 1DPF. **a** Venn diagram of all upstream regulatory molecules analyzed. **b** Heatmap of predicted activation z-scores for selected classes of upstream regulatory molecules. Green indicates predicted activation, blue indicates predicted inhibition, white indicates the regulator is not predicted to function in that organ, and grey indicates that the upstream regulator is predicted to have significant involvement but the activation state cannot be determined based on the gene expression data. Regulators shown in this heatmap were filtered by three conditions: 1) were present in at least three of the four organs, 2) are significantly predicted (*p*-value < 0.05), and 3) have activation z-scores greater than |1.5| in at least one organ. Biological drug, chemical, and microRNA categories were excluded from URM analyses

Our inferences of upstream regulatory molecules (URMs) between the fasted and 1 DPF time points supported many of the same molecular mechanisms underlying organ growth identified via CPA, such as stress response, growth, and lipid signaling pathways. We explored URM predictions for all classes of URMs except biological drugs, chemicals, and microRNAs. We found that many predicted URMs were shared among organs, with 51 shared among all four organs. Predicted URMs also showed substantial organ-specific patterns, with a large number of URMs uniquely predicted for each organ. The heart showed the largest number of unique URM predictions (269), while only 123, 167, and 137 unique URMs were predicted in the kidney, liver, and small intestine, respectively (Fig. 4a).

To identify regulators with broadly relevant patterns across multiple organs, we focused on URMs predicted significantly in at least three organs and with moderate to high activation z-score (z > |1.5|) in at least one organ. A subset of the URMs meeting these criteria is shown in Fig. 4b, and the full set is shown in Additional file 1: Figure S2. Many of these URM predictions coincided directly with predicted canonical pathways. NFE2L2 and ATF4, key regulators within the NRF2-mediated oxidative stress response pathway, were predicted to be strongly activated in the small intestine, liver, and kidney, consistent with the canonical pathway analysis predictions of activation of the overall NRF2 pathway in these three organs. We also predicted involvement of NFkB and NFkBIA, two key regulators within the NFkB signaling response pathway – this inflammatory response pathway is thought to be inhibited by activation of the NRF2-mediated oxidative stress response pathway [28, 29]. NFkB was predicted to be inhibited in the liver and heart, weakly activated in the kidney, and absent in the small intestine, while NFkBIA was predicted to be inhibited in the liver, weakly activated in the heart and kidney, and again absent in the small intestine. Activation of the growth pathways mTOR and PI3K/AKT were additionally supported by activation of predicted regulators such as mTORC1 and RAF1, respectively, and the inhibition of PTEN. Lipid signaling pathways such as LXR/RXR signaling, LPS/IL-1-mediated inhibition of RXR function, PPAR/RXR, and PPAR signaling were supported by several predicted URMs such as PXR ligand, NR1H3, NR1I2, NR1I3, SREBF1, SREBF2, PPARA, PPARG, RXRA, PPARGC1A, and PPARGC1B (Fig. 4). These URMs were consistently predicted as activated in the small intestine, liver, and kidney and either absent or predicted as inhibited in the heart.

It is notable that while the inferences of activation directions of lipid signaling pathways across organs were largely ambiguous and sometimes inconsistent in our CPA (Fig. 3), the associated URMs display a consistent trend of predicted activation in the small intestine, liver, and kidney, and either predicted inhibition or absence in the heart (Fig. 4). Additionally, several URMs, particularly for the mTOR pathway, were predicted as inconsistent or even contradictory to the results of CPA or our experimental data. For example, while mTORC1 is predicted as significantly activated in the small intestine by URMA, this molecule is downregulated in our experimental data (see Discussion for details). Additionally, the mTOR protein that is involved in forming both of the main complexes of the mTOR signaling pathway (mTORC1 and mTORC2) is predicted to be strongly inhibited in the small intestine and weakly inhibited in the kidney and heart. Both of these URM predictions appear to contradict the positive activation of the mTOR signaling pathway inferred for the small intestine and heart as inferred from the CPA.

In addition to URMs involved in key predicted canonical pathways, upstream regulatory analysis predicted several other notable URMs with strong activation or informative trends across organs. Insulin and INSR were both predicted as strongly activated regulators in the kidney, liver, and small intestine, suggesting a possible role of insulin receptor signaling in facilitating this regenerative response, which is also consistent with activation of the mTOR pathway. Myc, a regulator within the ERK5 and p38 MAPK signaling pathways, was predicted as activated in all four organs, although strongest in the liver. Several regulators within the MAPK signaling pathway were also predicted in URMA, with ATF4 and ATF6 predicted as activated in the kidney, liver, and small intestine, and ERK predicted as activated in the kidney and inhibited in the heart and liver. These URMs suggest the involvement of the ERK and MAPK signaling pathways in this response, even though CPA predictions for these two pathways were not substantially strong (Figs. 3 and 4).

### Detailed dissection of NRF2 and mTOR pathway responses to feeding

**Fig. 5:**
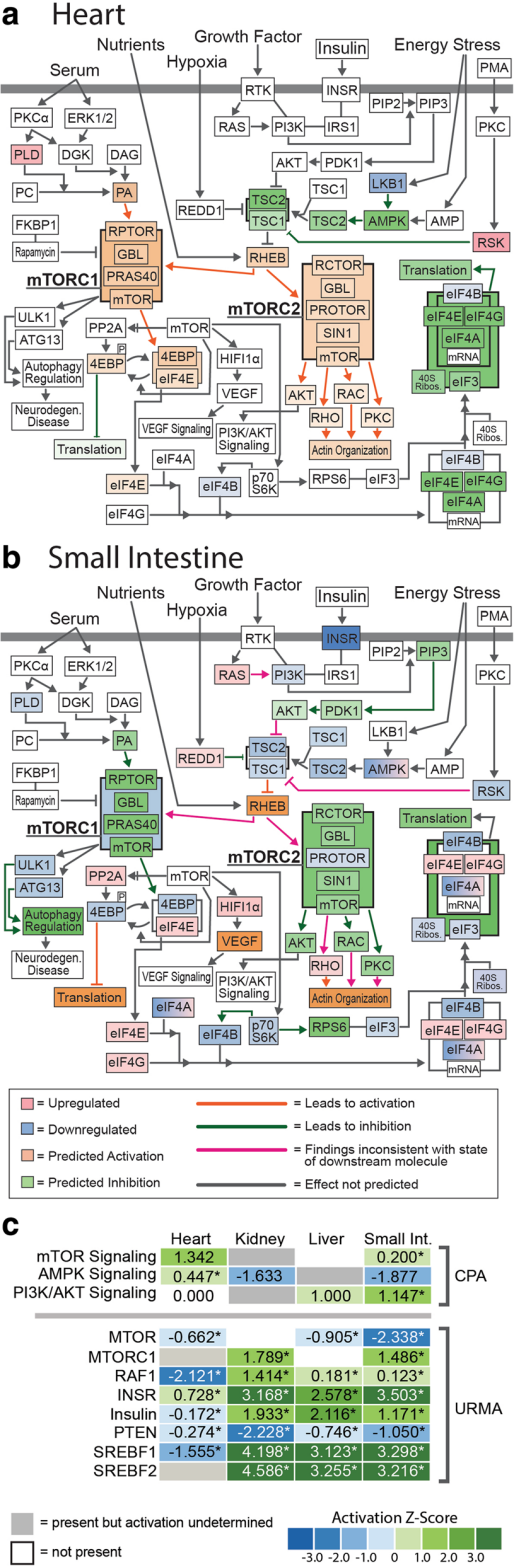
Combined gene expression and predicted activation information for the mTOR pathway in the heart and small intestine. **a** Gene expression and predicted activity for the mTOR pathway in the heart. **b** Gene expression and predicted activity for the mTOR pathway in the small intestine. Differentially expressed genes identified in our RNAseq data set are highlighted in red (upregulated) and blue (downregulated) while predicted activation states are highlighted in orange (activation) and green (inhibition). **c** CPA and URMA results for pathways and upstream regulatory molecules involved in mTOR signaling and other relevant growth pathways

**Fig. 6:**
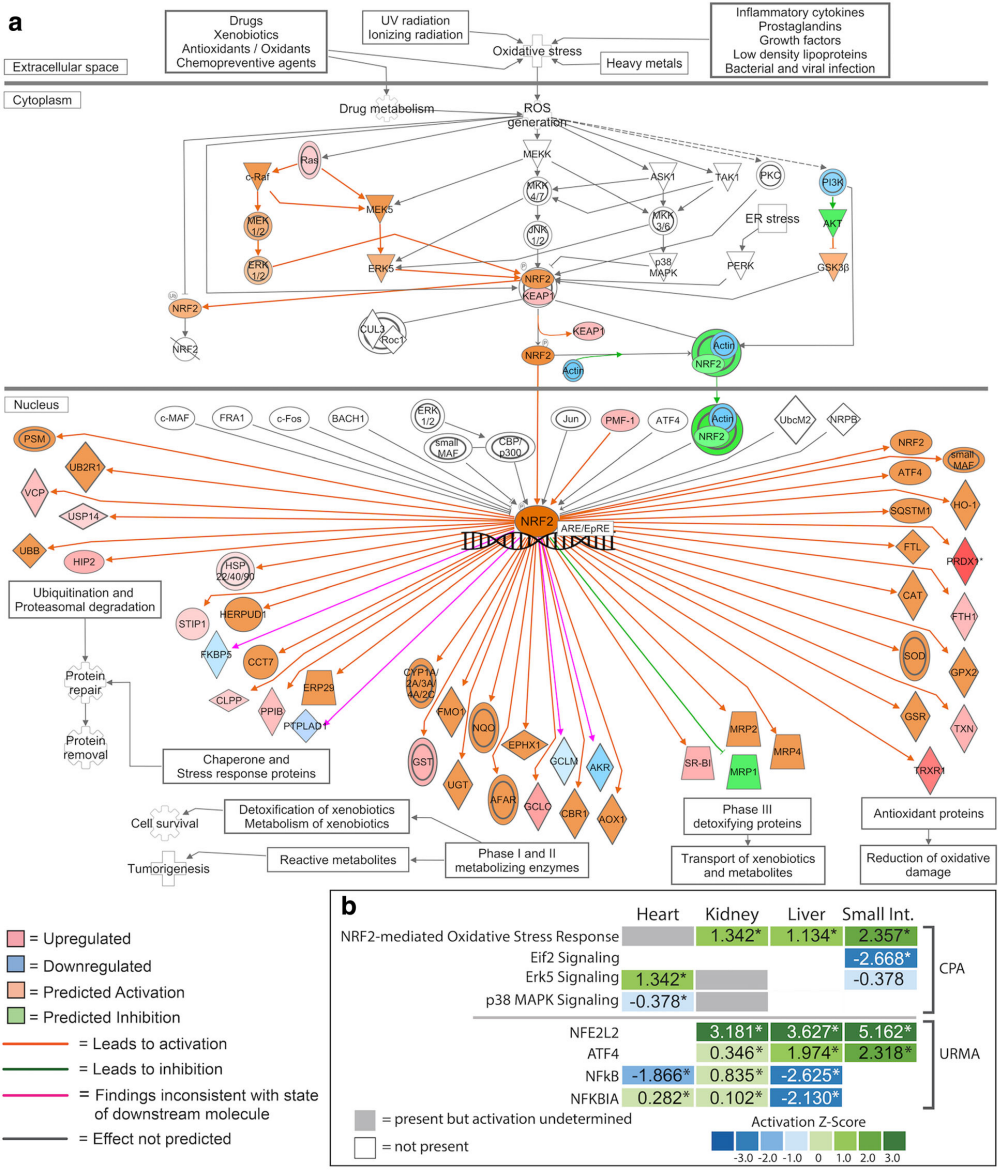
IPA generated pathway prediction for the NRF2-mediated oxidative stress response in the small intestine. Predicted activation state of the pathway was estimated using genes identified as significantly differentially expressed from our RNAseq data set. **a** Gene expression and predicted activity for the NRF﻿2 pathway in the small intestine. Differentially expressed genes identified in our RNAseq data set are highlighted in red (upregulated) and blue (downregulated) while predicted activation states are highlighted in orange (activation) and green (inhibition). **b** CPA and URMA results for pathways and upstream regulatory molecules involved in NRF2 signaling and other related pathways

We were particularly interested in our findings that the NRF2 stress response and the mTOR growth pathways appear to be involved in post-feeding growth in multiple organs. To investigate these inferences further, we fully dissected evidence from our gene expression data for activation of these pathways by visualizing observed and inferred evidence for activation of these pathways in the context of IPA-generated pathway maps (Figs. 5–6; Additional file 1: Figures S3–S6). Specifically, we generated pathway predictions that integrate both observed shifts in gene expression from our data (from fasting - 1DPF), and estimates of activation/inhibition of molecules downstream of these observed genes that are inferred based on canonical signaling patterns in these pathways. Relevant to our power to detect pathway-wide signals of activity, we were able to associate over 70% of human genes within the mTOR and NRF2 pathways with python orthologs that were expressed at some level in our dataset (see Additional file 1: Table S2); thus, we expect that our power and degree of resolution of pathway activation for these particular pathways is quite good.

Pathways maps for mTOR responsiveness between fasted and 1DPF show both common and divergent patterns of pathway activation among organs (Fig. 5 and Additional file 1: Figure S3). The heart (Fig. 5a) and kidney (see Additional file 1: Figure S3) both show similar patterns of mTOR activation, including the activation of both the mTORC1 and mTORC2 complexes. Major differences in mTOR activation between these two organs includes strong evidence for downregulation of AMPK and the eIF4 complex in the heart, yet, no direct and/or clear evidence for up- or downregulation of these complexes in the kidney. In the small intestine, the mTOR pathway was inferred to be strongly downregulated, as is AKT; AMPK and the eIF4 complex showed mixed signs of activation (both positive and negative) (Fig. 5b). It is also notable that different organs showed different levels of internal consistency in the integration of results with the known functionality within the mTOR pathway. For example, the heart and kidney have either zero or one pathway connection in which gene expression results contradict the direction of activation of the pathway (pink arrows in Fig. 5a and Additional file 1: Figure S3) – for the kidney this disagreement occurs in the relationship between RSK and inhibition of TSC1 (see Additional file 1: Figure S3). In the small intestine, eight such disagreements occur (Fig. 5b), and most of these occur at the steps immediately above or below activation of mTORC1 and mTORC2 complexes. The liver was the only organ that contained no signal for the activation or repression of mTOR pathway (i.e., no differentially expressed genes in this pathway were observed). It should be noted that inferences for mTOR activation from CPA are at times contradictory to those identified via URMA (Figs. 3–5; Additional file 1: Figure S3). While predictions based on the pathway maps indicate downregulation of mTOR in the small intestine, the z-score suggests slight upregulation of this pathway during regenerative growth in this tissue. URMA predicts inhibition of the mTOR molecule in the heart, kidney, and small intestine, while mTORC1 activation is predicted in both the kidney and small intestine, and undefined in the heart. Thus, while mTOR involvement in organ regenerative growth is clear across organs, the relationships between pathway scores, molecule-level inferences, and URMs are complex.

Pathway maps for the NRF2-mediated oxidative stress response between fasted and 1DPF indicate consistent activation of this pathway in the kidney, liver, and small intestine (Fig. 6; Additional file 1: Figures S4–S6). In addition to predicted responses inferred from CPA (Figs. 2 and 5; Additional file 1: Figures S4–S6), multiple observed genes in our dataset downstream of NRF2 are upregulated in these three organs, including *thioredoxin* (TXN), *glutathione s-transferase mu 1* (GST), and *peroxiredoxin 1* (PRDX1), providing confirmatory evidence of NRF2 activation. The response of this pathway in the heart is, however, less clear (see Additional file 1: Figure S4). In the heart, NRF2 responses were predicted based on the observed fold-change values of only four genes, and predictions suggest inhibition of this pathway in the heart (see Additional file 1: Figure S4) although the direction (activation versus inhibition) was not statistically significant (Fig. 3). It is also notable that we observed differences in the inferred consistency of integrated gene expression results and activation/inhibition inferences across organs (Fig. 6; Additional file 1: Figures S4–S6): in the heart, only two inconsistencies are observed while the kidney, liver, and intestine have one, two, or four inconsistencies, respectively. Inferences from URMA for the activation of NRF2 are highly consistent with activation inferences from CPA, including significant URM activation predicted for NFE2L1 in the liver and intestine and significant activation of NFE2L2 in kidney, liver, and small intestine (Fig. 4). In contrast, upstream regulators of this pathway were not predicted to be significantly activated or inhibited in the heart, inconsistent with the predictions given in the pathway figure (Fig. 4 and Additional file 1: Figure S4).

### Expression response between 1 and 4 DPF

In comparison to expression between fasting and 1DPF, the IPA analyses conducted on genes differentially expressed between 1DPF and 4DPF across organs predicted a substantially smaller number of pathways as significantly enriched, the majority of which were predicted with ambiguous directions of activation. This is likely due to the substantially smaller number of significantly differentially expressed genes identified in all organs between 1DPF and 4DPF, which is expected because 4DPF represents a sampling time intermediate between the peaking of organ growth and the regression of these phenotypes. This time interval (1DPF-4DPF) aimed to capture the early stages of organs shifting expression towards organ atrophy and towards a reversion to the fasted state, and we expected to observe partial reversals in pathways predicted to be active between fasted and 1DPF, and perhaps additional new pathways involved in apoptosis and atrophy. However, we found few consistent or clear patterns of interpretable pathway involvement between the 1DPF and 4DPF time points (see Additional file 1: Figure S7). Pathways predicted for this time interval include various pathways related to biosynthesis and stress response, such as unfolded protein response. We also inferred inconsistent involvement of these pathways across organs, and none were predicted with a direction of activation (see Additional file 1: Figure S7). Only one pathway, mitotic roles of polo-like kinase, was predicted as significant and with a direction of activation between 1DPF and 4DPF, and was predicted only in the small intestine. While we did infer a single lipid signaling pathway that also was indicated by CPA predictions from the fasted to 1DPF interval (LPS/IL-1 mediated inhibition of RXR function), the lack of predicted directions of activation and unclear involvement across organs prevents informative interpretation of the activity of this pathway between 1DPF and 4DPF. Collectively, these results suggest that the 4DPF time point may not be sufficient to capture shifts in gene expression that elucidate the mechanisms involved in the early stages of regression of organ phenotypes.

## Discussion

A detailed understanding of the molecular mechanisms capable of driving regenerative growth in vertebrates may provide important insights into the treatment of diverse human diseases. Because traditional vertebrate model systems offer limited insight into natural organ regenerative processes, non-traditional model systems, including snakes in general and Burmese pythons in particular, hold great potential for providing unique insights into vertebrate regenerative organ growth processes. In this study we have found that multiple integrated growth pathways, in addition to multiple stress-response pathways, appear to underlie the coordinated organ regenerative process in Burmese pythons upon feeding. Despite distinct patterns of gene expression associated with growth for each organ, pathway and upstream regulatory molecule analyses reveal substantial similarities in pathways associated with post-feeding, extreme-growth responses across multiple organs. Specifically, we found evidence for a consistent interactive role of three major types of pathways underlying growth responses in python organs following feeding, including the related growth pathways mTOR and PI3K/AKT, lipid-signaling pathways such as PPAR and LXR/RXR, and stress-response/cell-protective pathways including NRF2.

### mTOR and other growth pathways underlying organ growth

Across the four organs examined, we found evidence for the involvement of the mTOR signaling pathway as a key integrator of growth signals underlying post-feeding regenerative organ growth. This pathway integrates processes for the use of energy and nutrients to regulate growth and homeostasis [30]. mTOR interacts with multiple other pathways, including PI3K/AKT, several lipid metabolism and signaling pathways [30, 31], and the NRF2-mediated oxidative stress response [32, 33] – all of which are also active in multiple organs during growth (Figs. 3–5). mTOR complex 1 (mTORC1) is the most well-characterized of the two mTOR complexes and integrates signaling from growth factors, energy status, oxygen, and amino acids to promote cell growth when activated [31]. The TSC1/2 complex transmits upstream signals from growth factor and insulin signaling to modulate the activity of mTORC1 and its interaction with other pathways including PI3K/AKT [30, 31, 34]. The effector kinases of these external pathways inactivate the TSC complex through phosphorylation, thus, indirectly activating mTORC1 [30, 31]. AKT can also directly activate mTORC1 through phosphorylation of an mTORC1 inhibitor. In a low energy state, AMPK inhibits mTORC1 by phosphorylating regulatory associated protein of mTORC1 (RAPTOR) [30, 31]. mTORC2 signaling is less well-understood, but is known to respond to growth factors through PI3K signaling [30].

CPA of gene expression in the first 24 h after feeding indicate that involvement of the mTOR signaling pathway is significant in the small intestine (predicted activation), but insignificant in both the heart (predicted activation) and kidney (activation state undetermined). The liver lacked evidence of involvement of the mTOR signaling pathway from CPA (Figs. 3–4). In URM analysis, the mTOR molecule itself was predicted to be downregulated in the heart, liver, and intestine with no presence in the kidney, which contrasts our CPA results (Figs. 3–4). However, URMA-predicted activation of the mTORC1 complex is supported in both the kidney and small intestine with undefined involvement in the heart, and the liver shows no signal for mTORC1 (Fig. 4). Interestingly, CPA indicate mTORC1 is downregulated in the small intestine at 0–1DPF (Fig. 6), yet this downregulated state of mTORC1 is based only on the downregulation of a single gene, G protein subunit beta 1 like (GNB1L), which IPA identifies as a subunit of the mTORC1 complex. In contrast, AMPK signaling is predicted to be downregulated in the kidney and small intestine, indicative of elevated ATP levels and active mTORC1 [30, 31] (Fig. 3). It is notable that nearly all genes in the mTOR pathway were associated with python orthologs that were observed as expressed across our dataset (see Additional file 1: Table S2), which suggests that our inferences of non-responsive genes within the mTOR pathway are biologically meaningful (e.g., true negatives), rather than representative of a lack of data. Thus, mTOR signaling in python tissues during regenerative organ growth may include non-canonical features compared to typical models of mTOR signaling that account for the partial responsiveness of genes and targets inferred from our CPA.

Our results identify mTOR as a central regulator and integrator of a number of diverse growth signals that drive post-feeding regenerative organ growth in Burmese pythons. Insulin signaling represents a key-regulating factor of the mTOR pathway [31], and we found multiple lines of evidence indicating roles of insulin signaling in post-feeding growth responses. Specifically, 0–1DPF URMA inferred the activation of INSR and insulin, and the inhibition of INSIG1 and INSIG2, in the kidney, small intestine, and liver, and the inverse of these activation patterns in the heart. INSIG1 and INSIG2 are negative regulators of SCAP [35, 36], which in turn regulates SREBP activity. Consistent with inferences of inhibition of INSIG1–2, URMA predicted the upregulation of SREBF1 and SREBF2, which provide evidence of an increase in sterol-regulatory element activity coincident with organ growth [36, 37] (Fig. 4). In addition to the interaction of insulin signaling and mTOR activity, we also found multiple lines of evidence for PI3K/AKT signaling that would interact with mTOR. Our URMA indicates significant downregulation of PTEN, an upstream regulator of the PI3K/AKT pathway, across all four organs, and CPA predicts activation of the PI3K/AKT signaling pathway in the small intestine and liver.

Evidence from previous studies also support the role of mTOR, PI3K/AKT, and AMPK signaling mechanisms in python post-feeding growth, at least in the heart. Western blots of python cardiac tissue post-feeding support the inference of early activation of mTOR and PI3K/AKT pathways by demonstrating that phosphorylated AKT and MTOR proteins increase significantly in abundance between 12 and 24 h post-feeding [11]. These western blots also demonstrated phosphorylated AMPK protein was upregulated within 24 h post-feeding, but lagging temporally behind the peak in phosphorylated MTOR and AKT [11], consistent with the antagonistic relationship between AMPK and MTOR/AKT [30]. These independent lines of evidence for the roles of mTOR, PI3K/AKT, and AMPK signaling in python post-feeding organ growth confirm our inferences of the central roles of these pathways, and support the power of pathway and URM inferences for inferring signaling mechanisms.

MAPK and related pathways also appear to be prominently involved in organ growth responses post-feeding, which is sensible given their known interactions with multiple growth pathways, including PI3K/AKT signaling and mTOR [38–40]. Our data reveal the involvement of MAPK signaling most clearly in the heart, with significant enrichment and predicted inhibition of p38 MAPK signaling and significant activation of ERK5 signaling (Fig. 3). ERK5 is a member of the Mitogen-activated protein kinases (MAPKs) that is crucial to cell proliferation and activated in response to growth factors and oxidative stress [41, 42]. MYC is a downstream transcription factor regulated by the MAPK pathway and ERK5 specifically [43, 44], and an essential regulator of development and cell proliferation [45–47]. Our URMA predict significant activation of MYC in all four organs, indicating a broad role of active MAPK signaling in post-feeding organ growth in the python.

### NRF2 – protective function and interaction with growth pathways

One of the strongest and most consistent signals in the canonical pathway and upstream regulatory molecule analyses was the involvement of the NRF2-mediated oxidative stress response pathway. Commonly associated with anti-aging and longevity [48–50], injury repair, and mitigation of inflammation [51], evidence for the central involvement of the NRF2-mediated oxidative stress response pathway in the small intestine, liver, and kidney begs the question of whether there is an important yet largely unappreciated role for stress-response signaling pathways in growth responses, and regenerative organ growth in particular.

The NRF2 pathway was significantly upregulated in small intestine, kidney, and liver within the first day following feeding (Fig. 3), and the NRF2 transcription factor (NFE2L2) was one of the most significant and highest in magnitude URMs predicted in these three organs (*p*-values < 1.55e^-10^, z-scores > 3.0) (Fig. 4). The 24 h period following feeding in Burmese pythons involves unparalleled rates and magnitudes of organ growth, and also includes massive upregulation of metabolism – up to 44-fold increases in aerobic metabolism, which is among the highest fluctuation known for any vertebrate [3]. It is, therefore, sensible that activation of NRF2 is related to these major shifts in oxidative metabolism, and associated generation of reactive oxygen species [1, 2, 6, 9]. An open question, however, is what broader role the activation of NRF2 may play in facilitating the extraordinary growth responses associated with feeding in pythons. For example, post-fed Burmese python blood plasma has been shown to convey resistance to apoptosis to mammalian cells, even with exposure to high fatty acid concentrations that would otherwise cause cell death [11, 17]; such cell-protective qualities may be related to signals that activate NRF2 and/or other stress-response pathways. Interestingly, in addition to cell-protective roles of NRF2, this pathway also contains multiple points of integration with various growth pathways, including those activated in python organ regenerative growth.

The NRF2-mediated oxidative stress response pathway interacts with multiple pathways predicted in our canonical pathway analysis [52–57] (Figs. 3–4). The PI3K/AKT signaling pathway, predicted to be upregulated upon feeding in both the liver and small intestine, is essential for regulating the antioxidant functions of NRF2, and studies have shown that inhibition of this signaling pathway leads to attenuation of NRF2 activities [58, 59]. This interaction is evident when examining the role of NRF2 in the proliferation of cancer cells. Studies have shown that NRF2 is able to redirect glucose and glutamine into anabolic pathways through activation of PI3K/AKT signaling [60]. The activated PI3K/AKT pathway leads to greater accumulation of NRF2 in the nucleus, which allows NRF2 to enhance metabolic activities as well as promote cell proliferation and cytoprotection [60]. The PI3K/AKT signaling pathway activates mTOR activity in response to growth factors, and this and previous studies [11] have shown that PI3K/AKT and mTOR signaling are key growth pathways underlying organ regenerative growth in the Burmese python. Therefore, there appears to be strong and coordinated links between growth signaling (via PI3k/AKT and mTOR) and stress response signaling via NRF2 underlying organ growth in pythons following feeding. Like mTOR, a large majority of genes in the NRF2 pathway were associated with python orthologs and were observed as expressed across our dataset (see Additional file 1: Table S2), which indicates that our inferences of non-responsive genes within the NRF2 pathway are likely true negatives, rather than artifacts due to a lack of ortholog identification in the python. Accordingly, predicted but unobserved expression responses in the NRF2 pathway in pythons suggest that the absence of expected responses may represent novel or non-canonical aspects of python biology or of the organ regeneration response in pythons.

In addition to NRF2-mediated oxidative stress response, evidence for the involvement of other stress response signaling mechanisms in python post-feeding organ growth was also observed. EIF2 signaling, important in translational control and responsiveness to conditions of environmental stress [61, 62], is strongly downregulated in the intestine, yet, absent in the other three organs (Fig. 3). Acute phase response signaling, which is involved in restoring homeostasis following inflammation or injury [63], is predicted to be strongly downregulated in the liver and moderately upregulated (but non-significant in the heart; Fig. 3). The precise roles of these additional stress response mechanisms in regenerative organ growth in the python remains an open question, although there is strong and consistent signal for the involvement of multiple stress response pathways overall in python post-feeding organ growth.

### Role of lipid signaling in driving growth

Previous studies have shown evidence that molecules responsible for triggering python post-feeding organ growth circulate in the blood of the Burmese python [11, 64]. Riquelme et al. demonstrated that post-feeding python plasma was capable of inducing cardiomyocyte growth in pythons and mice, and that fasted python plasma supplemented with three particular fatty acids successfully stimulated cardiomyocyte growth in mice [11]. Because these fatty acids only facilitated a growth response in the presence of fasted Burmese python serum, it is likely that python plasma contains additional factors required for successful post-feeding regenerative growth and that fatty acids are only partially responsible for stimulating growth responses. In the heart, we found significant enrichment and predicted activation for the LXR/RXR activation pathway as well as predicted activation of this pathway (although insignificant enrichment with *P* > 0.01) in the small intestine (Fig. 3). LXR is a potent activator of the SREBP-1c gene [65], and our data predict clear and significant activation of both SREBF1 and SREBF2 upon feeding in the kidney, liver, and small intestine with significant downregulation and undefined direction for SREBF1 and SREBF2 in the heart, respectively (Fig. 4). When activated, these proteins directly enhance genes important for the uptake and synthesis of various lipids. SCAP, important for the activation of these SREB molecules, is also predicted to be strongly activated in the kidney, liver, and small intestine (Fig. 4) [35, 36, 66].

We also examined PPAR signaling as a potential pathway for lipid signaling during this regenerative growth, given the central role of PPAR in mediating fatty acid signaling as well as its effects on gene expression [67]. PPAR has also been identified as an important regulator of cell survival during wound repair and regeneration [68]. Although CPA did not detect significant PPAR signaling activation, URMA significantly predicted PPARA, PPARG, PPARGC1A, and PPARGC1b involvement across organs, typically inhibited in the heart and activated in the other three organs in 0–1DPF comparisons (Fig. 4). Given the variations in pathway and URM inferences between the heart and the other three organs, the question of whether fatty acids also play a similar stimulatory role in regenerative growth in the small intestine, liver, and kidney as they do in the heart remains. Our results do, however, argue for a poorly understood yet central role of lipid-signaling in these growth responses, and suggest that the unusually strong bioactivity of fatty acids may elicit growth through conserved canonical pathway signaling mechanisms.

### Early phases of organ regression following digestion

Physiological studies have shown that python post-feeding organ growth peaks between 1DPF and 3DPF [1, 2, 5, 9] and that phenotypes begin to decline by 4DPF [2, 3, 7, 9]. Thus, as post-feeding growth phenotypes reverse from 1DPF to 4DPF, we expected to observe shifts towards the fasted state, such as the reversal or inhibition of growth-associated pathways. Relative to comparisons between fasting and 1DPF, comparisons between 1DPF and 4DPF yielded nearly an order of magnitude fewer significantly differentially expressed genes (Table 1). Accordingly, expression heatmaps (Fig. 2) and expression profile summaries (see Additional file 1: Figure S1) show that expression profiles of many genes at 4DPF tend to remain elevated (i.e., similar to levels at 1DPF), or exist at intermediate levels (between fasted and 1DPF levels of expression). We did not observe any particularly informative trends in canonical pathways and upstream regulator molecule predictions (see Additional file 1: Figure S7) associated with shifts in gene expression from 1DPF to 4DPF, and this result is not surprising given the relatively small number of genes that significantly change between these time points. Among the predicted pathways were several that are related to stress response and biosynthesis (see Additional file 1: Figure S7), although a lack of predicted direction of activation prevents detailed interpretation of the involvement of nearly all pathways predicted between 1DPF and 4DPF. The only pathway predicted as significant and with a direction of activation between 1DPF and 4DPF was the mitotic roles of polo-like kinase pathway, which was activated in the small intestine (see Additional file 1: Figure S7). It therefore remains an open question whether atrophy and other processes involved in reverting to the fasting state are controlled actively (via a new signal that stimulates the apoptotic and atrophy processes), passively (the signal (s) that stimulates the initial cascade of responses fades or stops), or some combination of the two mechanisms. Collectively, our results suggest that comparisons between the 1DPF to 4DPF time points may not be sufficient to predict the physiological mechanisms involved in phenotypic regression with adequate power. Further experiments, possibly with multiple later-stage time point sampling, may be required to address outstanding questions about how these growth phenotypes are reversed.

### Comparison of python organ regeneration to other regenerative model systems

Organ regeneration in snakes represents an extreme and unique phenotype among vertebrates. However, other examples of regenerative growth do exist among vertebrates, such as limb regeneration in salamanders [69], fin regeneration in fish [70], and regenerative heart growth in zebrafish [71, 72] and prenatal mammals [73]. This begs the question of whether or not these regenerative responses share common mechanisms, and as we continue to better understand the mechanisms driving regenerative growth in snakes, such key comparisons can begin to be made. While none of these other vertebrate regenerative growth systems directly parallel regenerative organ growth in snakes, regeneration of heart tissue in zebrafish is the most analogous comparison, as it occurs in adult organisms and represents regenerative growth of organ tissue specifically. Following injury or amputation of cardiac tissue, zebrafish hearts grow primarily by dedifferentiation and subsequent proliferation of cardiomyocytes [72]. Conversely, python hearts grow only by hypertrophy [3, 11, 74], and therefore may be driven by largely different regenerative mechanisms. The python small intestine, liver, and kidney, however, do grow via by hypertrophy and hyperplasia [3, 5, 11, 12]; while they represent different organ systems than the zebrafish heart, they may be driven by similar pathways that regulate cell proliferation in general. Indeed, there are parallels between zebrafish and python responses in the shared involvement of p38 MAPK signaling, a negative regulator of cardiomyocyte proliferation in zebrafish [71] that we infer to be inhibited in the Burmese python heart between fasting and 1DPF (Fig. 3). Additionally the mitotic roles of polo-like kinase pathway, which was the only pathway we predicted as significant and with a direction of activation between 1DPF and 4DPF (activated in the small intestine; see Additional file 1: Figure S7) is also involved in zebrafish regenerative heart growth. Cell-cycle regulation by polo-like kinase 1 is an important component of cardiomyocyte proliferation in zebrafish [72], and therefore may be playing a similar role in the python small intestine, although it is notable that it was not predicted as significant between fasting and 1DPF, when growth is presumably greatest in this organ [3, 5]. Other pathways involved in zebrafish regenerative growth, such as IGF signaling, FGF signaling, HIPPO signaling, and TGF-Beta signaling [71], were not inferred as significant based on canonical pathway analyses of either post-feeding time interval in our study of the Burmese python. TGFB1 and IGF1 growth factors were, however, inferred in our URMA analysis of the fasting to 1DPF interval (see Additional file 1: Figure S2), suggesting that there may still be some involvement of these growth factors in the regulation of regenerative growth in the Burmese python. A key conclusion based on our study is that, to our knowledge, mTOR signaling and NRF2-mediated oxidative stress response pathways have not been implicated in zebrafish regenerative growth. Thus, regenerative organ growth in the Burmese python appears to remain quite unique among vertebrates, both in the nature of the phenotype, and now in the molecular mechanisms underlying growth.

## Conclusions

Multiple coordinated growth pathways appear to play an important role in facilitating regenerative organ growth in multiple tissues of the Burmese python, and the overlap of pathways across organs suggests common signaling molecules may drive this response – consistent with evidence that common factors circulating in the plasma of pythons are capable of eliciting growth [11, 64]. Our analyses provide strong evidence for the involvement of particular growth and stress response pathways in post-feeding organ growth responses in multiple organs, although it is notable that our inferences of the activation versus inhibition of mechanisms was not always consistent across analyses (e.g., CPA versus URMA). As discussed above, such conflicting inferences could be due to the fundamental differences in CPA and URMA (e.g., n), in that they are integrating very different sources of evidence, coupled with the possibility that the continuous nature of this response may survey various mechanisms during an inflection point of activity that can confound inferences of directionality. However, contradictory inferences of mechanistic activation may also suggest that some of these core signaling pathways function differentially in snakes, or that some molecules or pathways are signaling via non-canonical mechanisms. Experiments have demonstrated that exposure to Burmese python 2DPF blood serum elicits significant growth of rat cardiomyocytes [11], as well as increases in size and insulin production of human pancreatic beta cells [17]. These findings suggest that even if regenerative organ growth in snakes is achieved in part by non-canonical pathway or regulator activity, core aspects of signaling underlying organ growth in pythons is conserved across vertebrates. Among the most intriguing results of this study is the consistent predicted activation of the NRF2-mediated oxidative stress response pathway, and NRF2-related signaling molecules, during regenerative organ growth. The integration of NRF2 signaling with other growth pathways, including mTOR, provide an exciting and novel mechanistic hypothesis for how NRF2 and other stress-response pathways may play an important yet largely unappreciated role in regenerative growth responses in vertebrates.

## Additional file

Additional file 1: Figure S1. STEM analysis of all genes differentially expressed across all time points (fasted – 4DPF). All significant expression profiles are shown with *P*-value and number of genes following that profile. **Figure S2.** Heat maps depicting activation z-scores for classes of upstream regulator molecules. Green indicates predicted activation, blue indicates predicted inhibition, white indicates that the regulator is not predicted to function in that organ, and grey indicates that the upstream regulator is predicted to have significant involvement but the activation state cannot be determined based on the gene expression data. Regulators shown on the heat maps were filtered by activation z-scores greater than |1.5| in at least one tissue. **Figure S3.** Combined gene expression and predicted activation information for the mTOR pathway in the kidney. Differentially expressed genes identified in our RNAseq data are highlighted in red (upregulated) and blue (downregulated) while predicted activation states are highlighted in orange (activation) and green (inhibition). **Figure S4.** Pathway prediction for the NRF2-mediated oxidative stress response in the heart. Predicted activation state of the pathway was estimated using genes identified as significantly differentially expressed from our RNAseq data set. **Figure S5.** Pathway prediction for the NRF2-mediated oxidative stress response in the kidney. Predicted activation state of the pathway was estimated using genes identified as significantly differentially expressed from our RNAseq data set. **Figure S6.** Pathway prediction for the NRF2-mediated oxidative stress response in the liver. Predicted activation state of the pathway was estimated using genes identified as significantly differentially expressed from our RNAseq data set. **Figure S7.** Pathway analysis of gene expression responses from 1DPF to 4DPF. Bar graph showing significant canonical pathways (Fisher’s Exact test *P* < 0.01) enriched for genes differentially expressed at these time points. Pathways were filtered to include those with at least one significant *p*-value in one of the four organs. Bars are colored based on the predicted activation Z-score for that pathway. **Table S1.** Sequencing information for all included python samples. PE76 and PE120 stand for the sequence read type (e.g., Paired-end 76 bp). The year provided represents the year in which the sample was sequenced. **Table S2.** The number of genes involved in each pathway as defined by IPA, the number of genes in the pathway that were assigned python orthologs via tblastx, and the number of those python orthologs observed with a non-zero level of expression in our dataset. (PDF 4804 kb)

## Abbreviations

CPA: Canonical pathway analysis
DPF: Days post-feeding
IPA: Ingenuity pathway analysis
URMA: Upstream regulatory analysis
