# Supplementary material for: Growth and stress response mechanisms underlying post-feeding regenerative organ growth in the Burmese python
Source: BMC Genomics. 2017 May 2;18:338. doi: 10.1186/s12864-017-3743-1 (PMC5412052; doi:10.1186/s12864-017-3743-1)
Supplement: Additional file 1: Figure S1. — STEM analysis of all genes differentially expressed across all time points (fasted – 4DPF). All significant expression profiles are shown with P-value and number of genes following that profile. Figure S2. Heat maps depicting activation z-scores for classes of upstream regulator molecules. Green indicates predicted activation, blue indicates predicted inhibition, white indicates that the regulator is not predicted to function in that organ, and grey indicates that the upstream regulator is predicted to have significant involvement but the activation state cannot be determined based on the gene expression data. Regulators shown on the heat maps were filtered by activation z-scores greater than |1.5| in at least one tissue. Figure S3. Combined gene expression and predicted activation information for the mTOR pathway in the kidney. Differentially expressed genes identified in our RNAseq data are highlighted in red (upregulated) and blue (downregulated) while predicted activation states are highlighted in orange (activation) and green (inhibition). Figure S4. Pathway prediction for the NRF2-mediated oxidative stress response in the heart. Predicted activation state of the pathway was estimated using genes identified as significantly differentially expressed from our RNAseq data set. Figure S5. Pathway prediction for the NRF2-mediated oxidative stress response in the kidney. Predicted activation state of the pathway was estimated using genes identified as significantly differentially expressed from our RNAseq data set. Figure S6. Pathway prediction for the NRF2-mediated oxidative stress response in the liver. Predicted activation state of the pathway was estimated using genes identified as significantly differentially expressed from our RNAseq data set. Figure S7. Pathway analysis of gene expression responses from 1DPF to 4DPF. Bar graph showing significant canonical pathways (Fisher’s Exact test P < 0.01) enriched for genes differentially expressed at these time points. [file 12864_2017_3743_MOESM1_ESM.pdf]

Supplementary Methods

Feeding Experiments

The following information pertains to snakes that were sampled and sequenced for this study (see also Additional file 1, Table S1; and [5, 13] for details regarding previously sequenced data incorporated in this study). Burmese pythons (*Python molurus bivittatus*) were purchased within 1-2 months of hatching from commercial vendors. All snakes included in this study originated from captive colonies, were phenotypically normal in coloration (i.e., no albino animals), and ranged in age from 9 months to 6 years (mean = 1.9 years) and in mass from 406 to 5,776 grams (mean = 1,036 g). Snakes were housed individually in 12L plastic bins that slide into customized racks in the Central Animal Care Facility at the University of Alabama. Each bin featured a floor substrate of newspaper and contained a water bowl. All pythons were maintained on a light/dark cycle of 14 hours of light followed by 10 hours of dark. Room temperature was maintained at 26-28°C and was constantly monitored by the Central Animal Care Facility. Prior to experimentation, pythons were fed weekly a meal of 1-2 rodents (adult mice or small rats) and water was provided *ad libitum*. Pythons were monitored daily by the Animal Care staff and personnel of the laboratory of Dr. Stephen Secor prior to and during experimentation. There were no interventions in snake care prior to or during experimentation. All experimentation and dissection was performed by Secor lab personnel. No special attention was given to selecting animals randomly from a research colony, however there was an attempt for matching in sexes (7 males: 6 females), so that there would be no bias due to sex in any treatment or the experiment overall. At the time of sampling, all animals were in good health and had not been subjected to any previous procedures or drug administration. Fasted snakes had been fasted for a minimum of 30 days prior to sampling. Snakes of the 1 and 4 days post-feeding treatments had been fasted for 30 days and then fed a rodent meal equal in mass to 25% of the snake body mass, and sampled 1 and 4 days after feeding, respectively. The mean mass of snakes in each treatment were: fasted (1,504 g), 1DPF (892 g), and 4DPF (593 g). At the time of sampling, snakes were sacrificed by humanely severing the spinal cord immediately behind the head; this provided the most efficient and rapid means to obtain organ samples for storage and study without compromising physiological responses of interest. Organ tissues were immediately extracted, snap frozen in liquid nitrogen, and stored at -80°C. Feeding experiments and subsequent sampling of snakes were completed over a span of several years, with fasted snakes sampled in 2005 and 2009, and 1 and 4DPF snakes sampled in 2005 and 2006. There was no particular order to the sampling of tissues from animals. No adverse events occurred during animal care or experimentation, and thus no modifications to the experimental protocol were undertaken as a result.

The Burmese python has become an outstanding animal model (compared to traditional mammal model systems) to explore the cellular and molecular mechanisms underlying regenerative organ growth and physiology, and therefore serves as an excellent replacement for exploring such systems in typical mammalian models. We made efforts to minimize the number of animals used overall in this study, as evident in the relatively small sample sizes for each treatment (3-6 individuals). Additionally, dissection of snakes included the removal and storage of all organs and other tissues (muscle, blood, etc.) so that subsequent studies can

utilize these tissues to study this regenerative phenotype in other organ systems without the need for the sacrifice of additional animals.

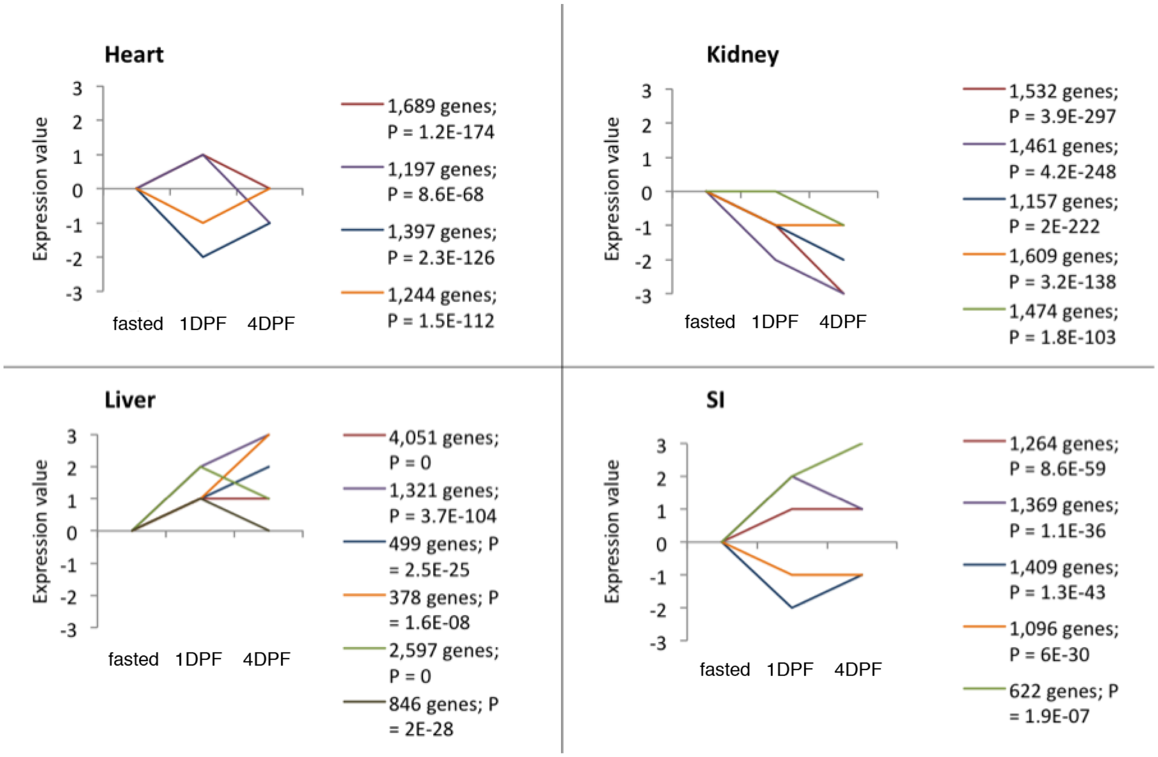

Figure S1. STEM analysis of all genes differentially expressed across all time points (fasted – 4DPF). All significant expression profiles are shown with P-value and number of genes following that profile.

Class	Regulator	Heart	Kidney	Liver	S.I.
Complex	CD3	-0.239	-2.525	-1.210	-2.218
Complex	NFkB	-1.866	0.835	-2.625	
Complex	LDL	-2.296	-0.635	-0.065	
Complex	MTORC1		1.789		1.486
Complex	PDGF BB		2.184	-1.649	1.013
Complex	PXR ligand...		0.277	1.446	1.913
Cytokine	IL1B	-1.739	1.601	-0.541	-1.089
Cytokine	IFNG	-1.031		-2.091	-1.576
Cytokine	IL5		1.772	2.839	1.341
Enzyme	POR	2.138	-3.484	0.000	-1.584
Enzyme	EVOLV5		-1.980		-1.477
Enzyme	KRAS	-0.093		2.744	2.529
Enzyme	FBXO32	-0.277		2.621	2.688
Enzyme	MGEA5	0.000	-1.807		-0.130
Enzyme	ACOX1		-1.066	-1.138	-2.083
Enzyme	GNA12		-0.200	-2.000	-1.786
Enzyme	CD38		-0.186	2.680	-0.175
Group	Insulin	-0.172	1.933	2.116	1.171
Group	ADRB	0.563	-3.051	-1.219	-1.671
Group	ERK	-1.082	1.254	-1.979	
Group	N-cor	1.067	-2.219		-1.195
Ion Channel	CFTR	-0.447	0.000	1.912	1.362
Ligand-dependent nuclear receptor	PPARA	-2.152	0.429	2.168	2.546
Ligand-dependent nuclear receptor	ESR1	-0.705	1.104	1.891	-1.355
Ligand-dependent nuclear receptor	PPARG	-3.173	2.041	0.603	3.273
Ligand-dependent nuclear receptor	NR1H3	-1.014	1.616	1.195	2.376
Ligand-dependent nuclear receptor	RXRA	0.186	1.353	2.549	1.027
Ligand-dependent nuclear receptor	PGR	-2.008		-0.399	0.595
Ligand-dependent nuclear receptor	NR1I2		1.665	1.627	1.449
Ligand-dependent nuclear receptor	ESRRA		2.611	0.905	2.800
Ligand-dependent nuclear receptor	NR1I3		0.651	1.715	1.496
Phosphatase	PTEN	-0.274	-2.228	-0.746	-1.050
Transcription regulator	MYC	1.270	1.660	4.721	1.347
Transcription regulator	PPARGC1A	-3.395	1.811	0.085	2.384
Transcription regulator	SP1	-1.353	2.845	0.671	0.305
Transcription regulator	HNF4A	-0.784	1.030	-0.014	3.083
Transcription regulator	PML	0.726	-2.099	-0.993	-1.723
Transcription regulator	SREBF1	-1.555	4.198	3.123	3.298
Transcription regulator	SREBF2		4.586	3.255	3.216
Transcription regulator	FOXO1	-1.562	0.243	-1.642	0.629
Transcription regulator	JUN	-0.203	1.894	-0.444	

Class	Regulator	Heart	Kidney	Liver	S.I.
Transcription regulator	SMARCA4	-2.159	1.726	-0.954	
Transcription regulator	NFKBIA	0.282	0.102	-2.130	
Transcription regulator	SMAD7	0.816		1.664	0.457
Transcription regulator	EPAS1	-1.644	-1.163		-0.156
Transcription regulator	CTNNB1	-2.155	0.239		-0.937
Transcription regulator	FOXO3	-1.803	-1.308		-0.800
Transcription regulator	NUPR1	-1.698	0.659		0.072
Transcription regulator	KLF15		1.880		2.232
Transcription regulator	PPARGC1B		3.278	0.130	3.049
Transcription regulator	NFE2L2		3.181	3.627	5.162
Transcription regulator	ATF4		0.346	1.974	2.318
Transcription regulator	XBP1		1.457	2.319	6.156
Transcription regulator	ATF6		0.394	2.186	1.543
Transcription regulator	FOXA3		-1.960		-0.059
Transcription regulator	MAFF				1.964
Transcription regulator	HNF1B		-0.555	-2.226	-0.027
Transcription regulator	NFE2L1			1.890	2.530
Transcription regulator	KDM5B		-0.514	-0.742	-1.812
Transporter	APOE	0.175		-2.190	
Transporter	ATP7B		3.317		3.317
Transporter	SLC13A1		-1.897	-2.236	-2.041
Other	INSIG1	2.383	-3.499	-1.362	-3.236
Other	INS	1.561	-0.915	1.567	0.158
Other	INSIG2		-2.768		-2.392
Other	NEDD9	-2.000			0.632
Other	SCAP		4.185	2.433	3.633
Other	INS1		1.881	-0.258	0.616
Other	GCG		0.277	-2.412	1.281
Growth factors	TGFB1	-1.022	2.617	-0.649	1.394
Growth factors	AGT	-0.763	2.263	-0.544	0.057
Growth factors	ANGPT2	-1.632		1.247	1.808
Growth factors	EGF		1.640	-1.051	2.827
Growth factors	INHA		-1.342	2.400	1.402
Growth factors	IGF1	-1.763	0.832	-0.224	
Kinase receptors	RAF1	-2.121	1.414	0.181	0.123
Kinase receptors	INSR	0.728	3.168	2.578	3.503
Kinase receptors	MTOR	-0.662		-0.905	-2.338
Kinase receptors	ZAP70		1.000	2.000	1.155
Transmembrane receptors	IL10RA	0.832	1.320	2.034	0.309
Transmembrane receptors	CD28	-0.674	-2.132	-0.955	-2.816

- Complex
- Cytokine
- Enzyme
- Group
- Ion Channel
- Ligand-dependent nuclear receptor
- Phosphatase
- Transcription regulator
- Transporter
- Other
- Growth factors
- Kinase receptors
- Transmembrane receptors

Activation Z-Score: -3.0 -2.0 -1.0 0 1.0 2.0 3.0

= present but activation undetermined

= not present

Figure S2. Heat maps depicting activation z-scores for classes of upstream regulator molecules significant between fasted and 1DPF. Green indicates predicted activation, blue indicates predicted inhibition, white indicates that the regulator is not predicted to function in that organ, and grey indicates that the upstream regulator is predicted to have significant involvement but the activation state cannot be determined based on the gene expression data. Regulators shown on the heat maps were filtered by activation z-scores greater than |1.5| in at least one tissue.

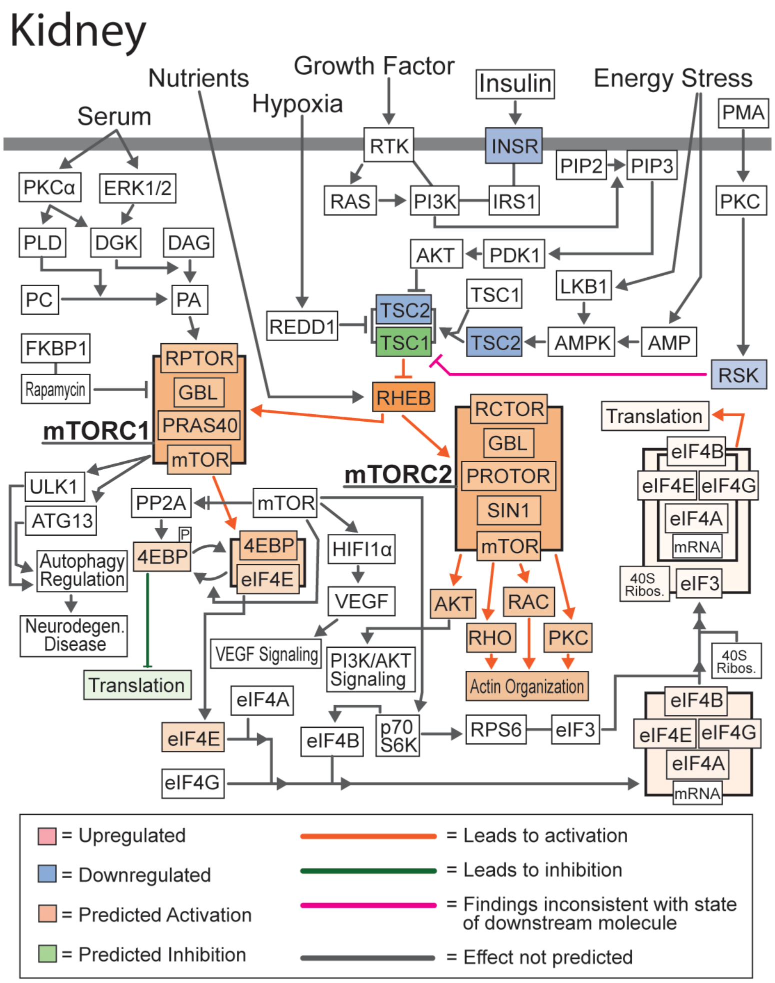

Figure S3. Combined gene expression and predicted activation information for the mTOR pathway in the kidney. Differentially expressed genes identified in our RNA-seq data are highlighted in red (upregulated) and blue (downregulated) while predicted activation states are highlighted in orange (activation) and green (inhibition).

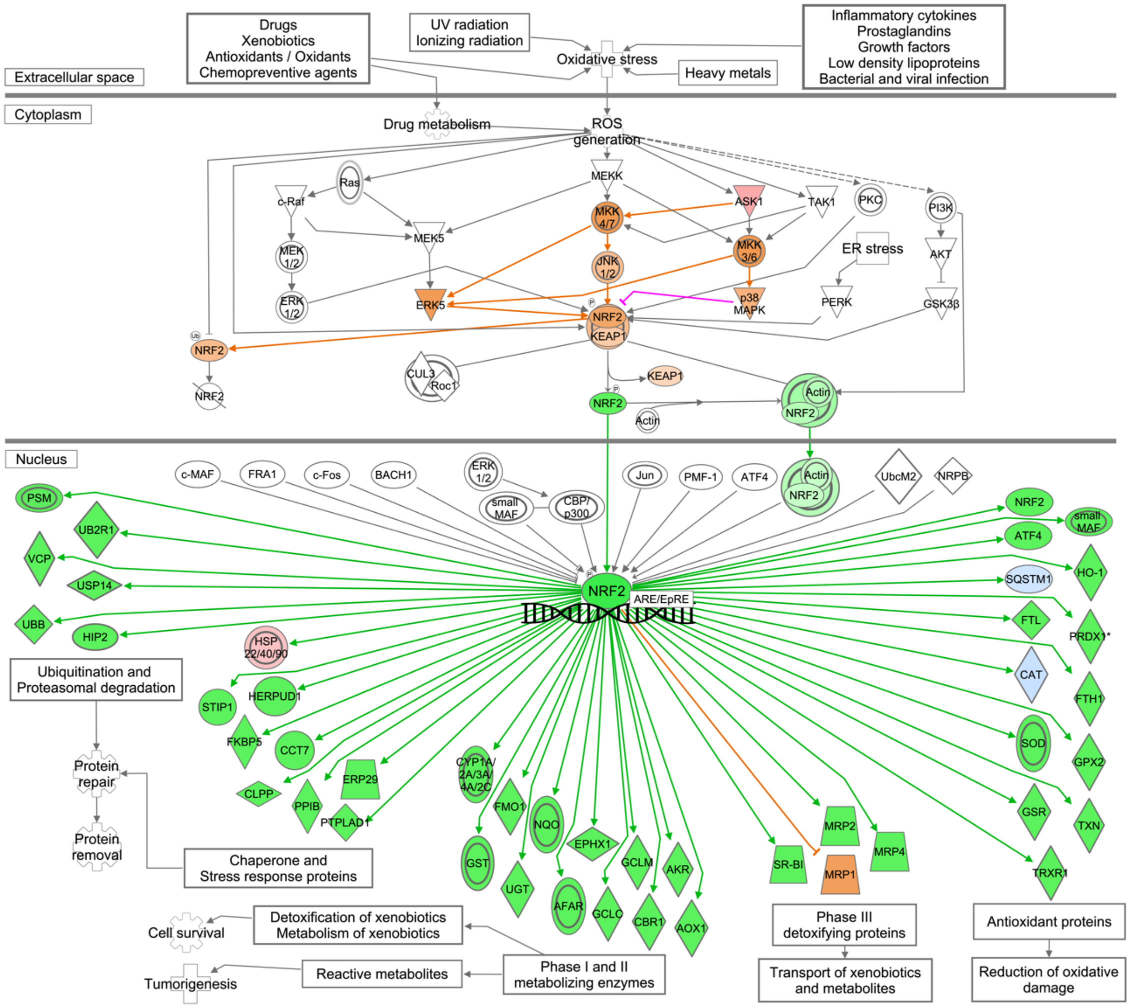

Figure S4. Pathway prediction for the NRF2-mediated oxidative stress response in the heart. Predicted activation state of the pathway was estimated using genes identified as significantly differentially expressed form our RNA-seq data set.

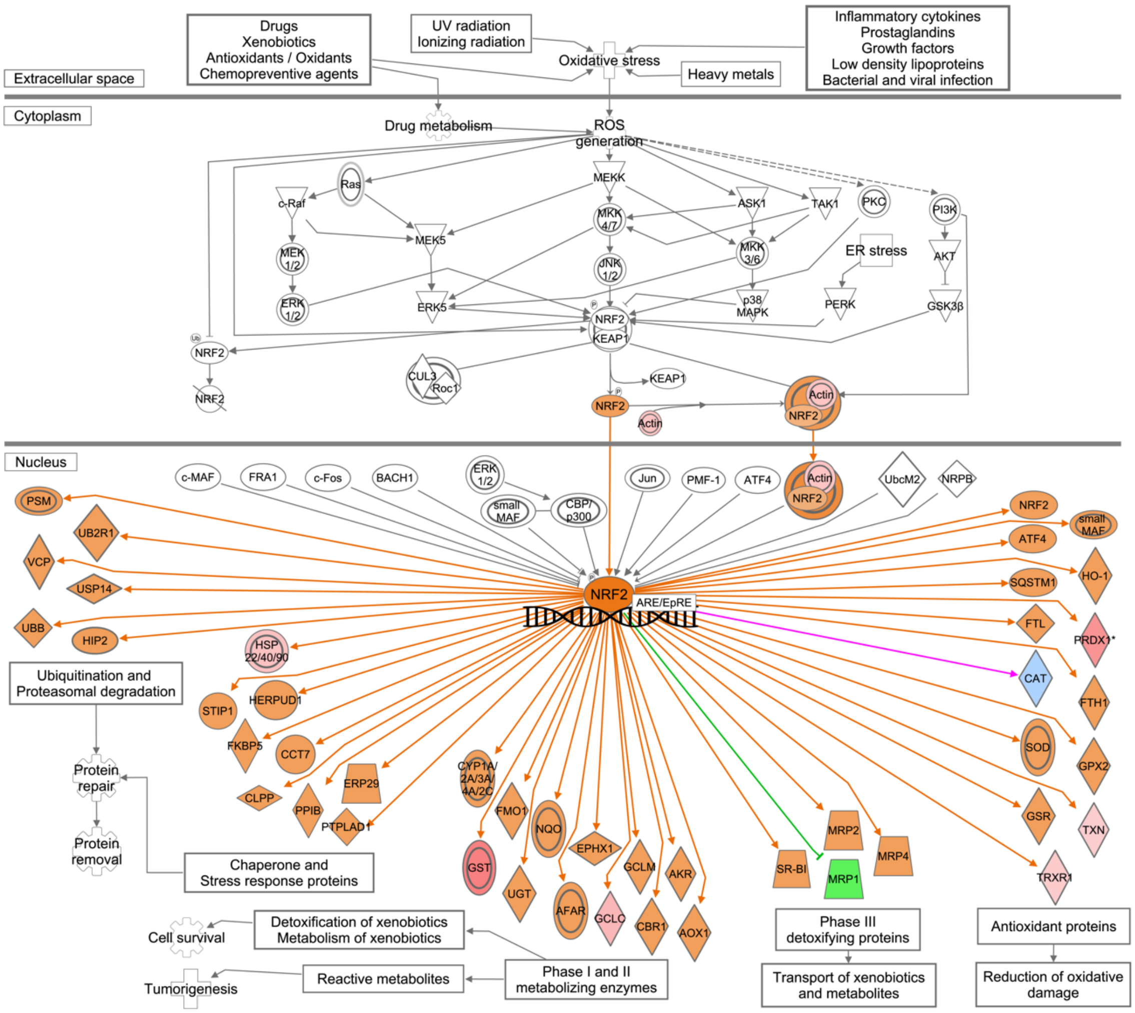

Figure S5. Pathway prediction for the NRF2-mediated oxidative stress response in the kidney. Predicted activation state of the pathway was estimated using genes identified as significantly differentially expressed from our RNA-seq data set.

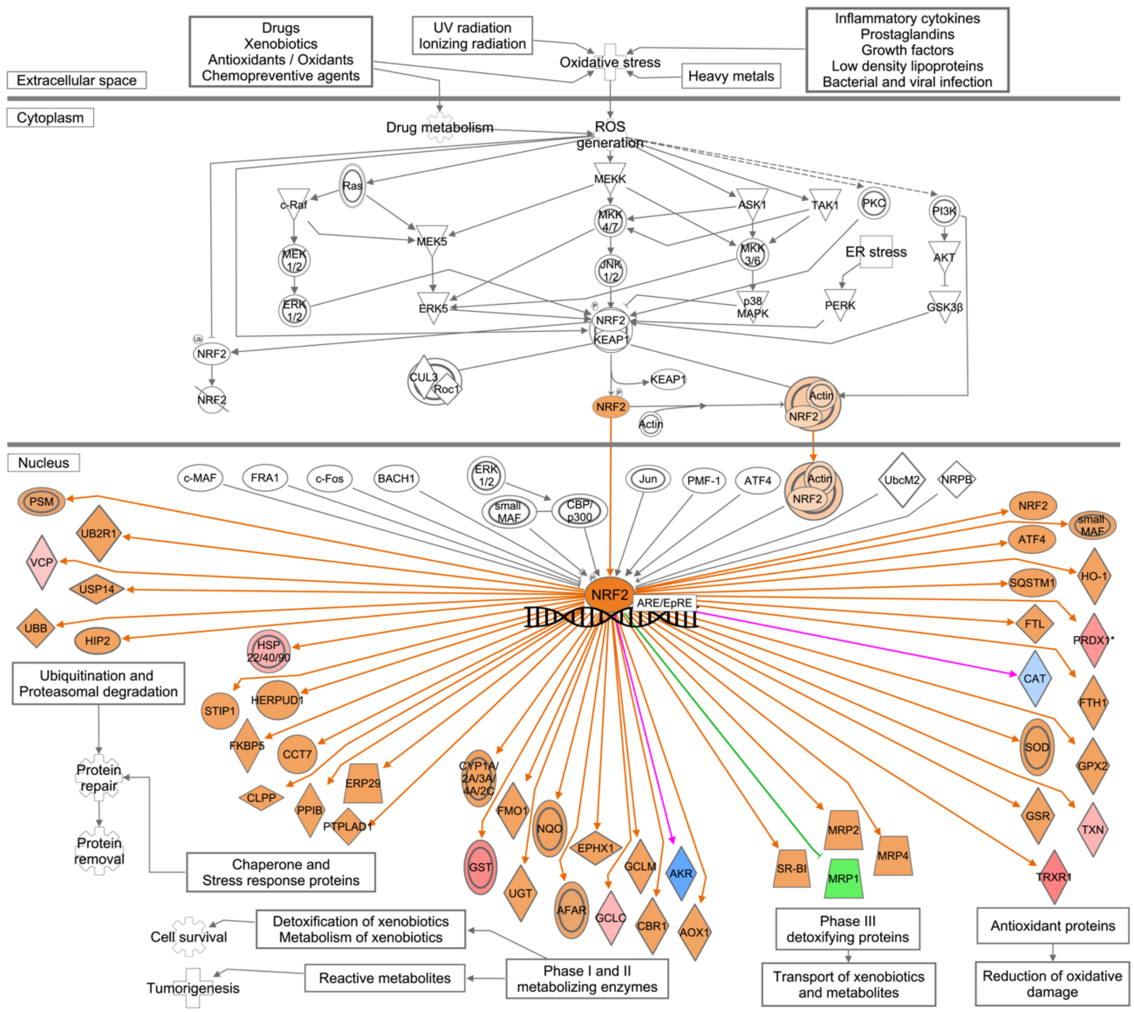

Figure S6. Pathway prediction for the NRF2-mediated oxidative stress response in the liver. Predicted activation state of the pathway was estimated using genes identified as significantly differentially expressed from our RNA-seq data set.

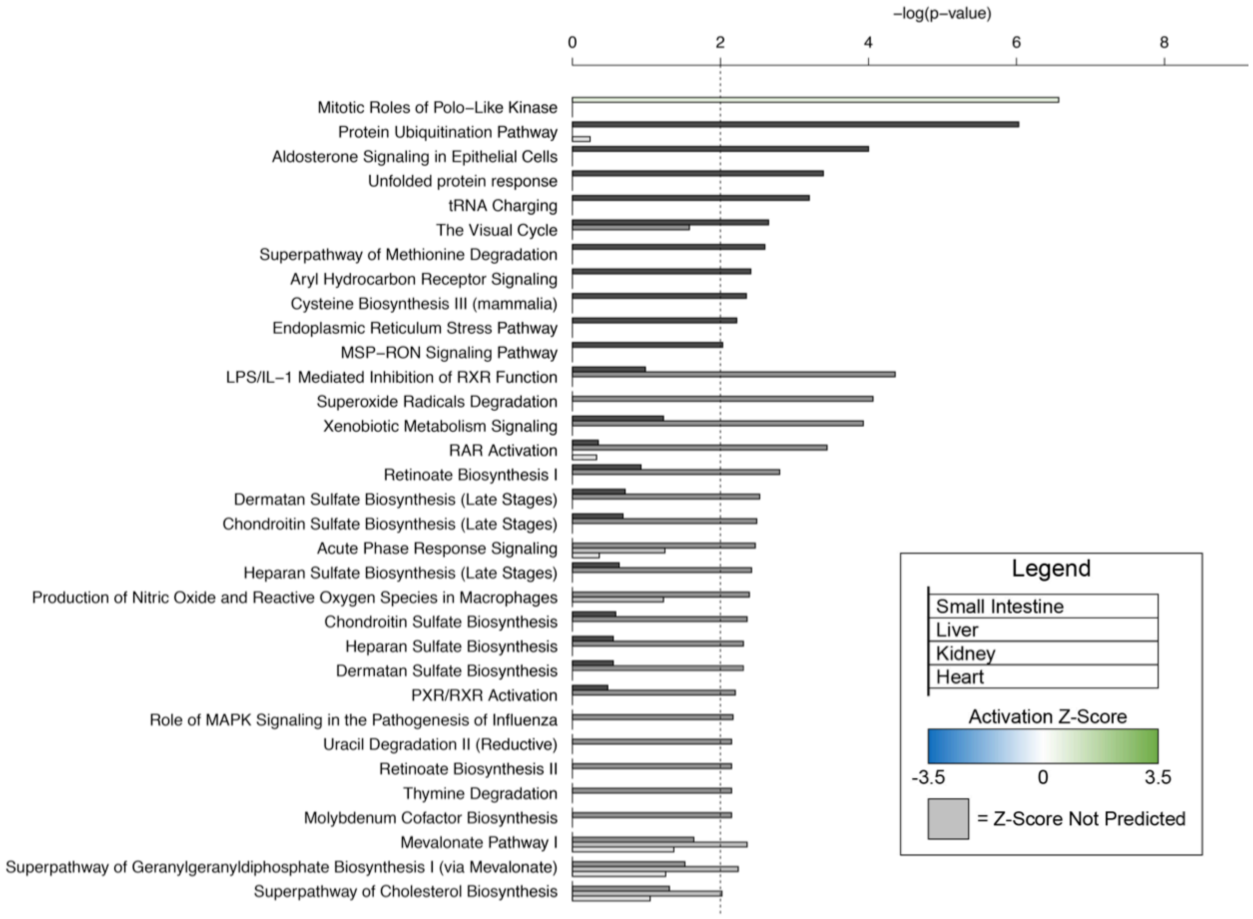

Figure S7. Pathway analysis of all genes significantly differentially expressed from 1DPF to 4DPF in the four organs. Bar graph showing significant canonical pathways (Fisher's Exact test P<0.01) enriched for genes differentially expressed at these time points. Pathways were filtered to include those with at least one significant p-value in one of the four organs. Bars are colored based on the predicted activation Z-score for that pathway.

Table S1. Sequencing information for all included python samples. PE76 and PE120 stand for the sequence read type (e.g., Paired-end 76bp). The year provided represents the year in which the sample was sequenced.

Tissue	Timepoint	Animal ID	Instrument	cDNA prep kit	Year	Sequence type	Library Name
Heart	fasted	AI6_1	GAIIx	Illumina Truseq	2010	PE76	TC01
Heart	fasted	AI6_2	GAIIx	Illumina Truseq	2011	PE120	TC05
Heart	fasted	AI11	GAIIx	Illumina Truseq	2010	PE76	TC01
Heart	fasted	AI8	HiSeq	NEB Next	2013	SE50	pRNA-A
Heart	fasted	U25	HiSeq	NEB Next	2013	SE50	pRNA-B
Heart	1DPF	Z12	GAIIx	Illumina Truseq	2010	PE76	TC01
Heart	1DPF	Z14_1	GAIIx	Illumina Truseq	2010	PE76	TC01
Heart	1DPF	Z14_2	GAIIx	Illumina Truseq	2011	PE120	TC05
Heart	1DPF	Z18	GAIIx	Illumina Truseq	2010	PE76	TC01
Heart	4DPF	Y5_1	GAIIx	Illumina Truseq	2010	PE76	TC01
Heart	4DPF	Y5_2	GAIIx	Illumina Truseq	2011	PE120	TC05
Heart	4DPF	Y18	GAIIx	Illumina Truseq	2010	PE76	TC01
Heart	4DPF	Y23	GAIIx	Illumina Truseq	2010	PE76	TC01
Kidney	fasted	AI8	HiSeq	NEB Next	2013	SE50	pRNA-A
Kidney	fasted	U25	HiSeq	NEB Next	2013	SE50	pRNA-B
Kidney	fasted	AI6_1	HiSeq	Illumina Truseq	2011	SE50	s1
Kidney	fasted	AI6_2	GAIIx	Illumina Truseq	2011	PE120	SP03
Kidney	fasted	AI11_1	HiSeq	Illumina Truseq	2011	SE50	s1
Kidney	fasted	AI11_2	GAIIx	Illumina Truseq	2011	PE120	SP03
Kidney	fasted	AJ6_1	HiSeq	Illumina Truseq	2011	SE50	s1
Kidney	fasted	AJ6_2	GAIIx	Illumina Truseq	2011	PE120	SP03
Kidney	fasted	AJ6_3	GAIIx	Illumina Truseq	2011	PE120	TC05
Kidney	1DPF	Z12_1	HiSeq	Illumina Truseq	2011	SE50	s1
Kidney	1DPF	Z12_2	GAIIx	Illumina Truseq	2011	PE120	SP03
Kidney	1DPF	Z14_1	HiSeq	Illumina Truseq	2011	SE50	s1
Kidney	1DPF	Z14_2	GAIIx	Illumina Truseq	2011	PE120	SP03
Kidney	1DPF	Z18_1	HiSeq	Illumina Truseq	2011	SE50	s1
Kidney	1DPF	Z18_2	GAIIx	Illumina Truseq	2011	PE120	SP03
Kidney	1DPF	Z18_3	GAIIx	Illumina Truseq	2011	PE120	TC05
Kidney	1DPF	V43	HiSeq	NEB Next	2013	SE50	pRNA-B
Kidney	1DPF	Z14_3	HiSeq	NEB Next	2013	SE50	pRNA-B
Kidney	4DPF	Y18_1	HiSeq	NEB Next	2013	SE50	pRNA-B
Kidney	4DPF	Y24	HiSeq	NEB Next	2013	SE50	pRNA-A
Kidney	4DPF	Y5_1	HiSeq	Illumina Truseq	2011	SE50	s1
Kidney	4DPF	Y5_2	GAIIx	Illumina Truseq	2011	PE120	SP03
Kidney	4DPF	Y5_3	GAIIx	Illumina Truseq	2011	PE120	TC05
Kidney	4DPF	Y18_2	HiSeq	Illumina Truseq	2011	SE50	s1

Kidney	4DPF	Y18_3	GAIIx	Illumina Truseq	2011	PE120	SP03
Kidney	4DPF	Y23_1	HiSeq	Illumina Truseq	2011	SE50	s1
Kidney	4DPF	Y23_2	GAIIx	Illumina Truseq	2011	PE120	SP03
Liver	fasted	AI6_1	GAIIx	Illumina Truseq	2010	PE76	TC01
Liver	fasted	AI6_2	GAIIx	Illumina Truseq	2011	PE120	TC05
Liver	fasted	AI8	HiSeq	NEB Next	2013	SE50	pRNA-A
Liver	fasted	AI11	HiSeq	NEB Next	2013	SE50	pRNA-B
Liver	fasted	U25	HiSeq	NEB Next	2013	SE50	pRNA-B
Liver	1DPF	V43	HiSeq	NEB Next	2013	SE50	pRNA-B
Liver	1DPF	Z14	HiSeq	NEB Next	2013	SE50	pRNA-A
Liver	1DPF	Z18	HiSeq	NEB Next	2013	SE50	pRNA-B
Liver	1DPF	Z12_1	GAIIx	Illumina Truseq	2010	PE76	TC01
Liver	1DPF	Z12_2	GAIIx	Illumina Truseq	2011	PE120	TC05
Liver	4DPF	Y5_1	GAIIx	Illumina Truseq	2010	PE76	TC01
Liver	4DPF	Y5_2	GAIIx	Illumina Truseq	2011	PE120	TC05
Liver	4DPF	Y18	HiSeq	NEB Next	2013	SE50	pRNA-B
Liver	4DPF	Y23	HiSeq	NEB Next	2013	SE50	pRNA-B
Liver	4DPF	Y24	HiSeq	NEB Next	2013	SE50	pRNA-A
Small intestine	fasted	AI8	HiSeq	NEB Next	2013	SE50	pRNA-A
Small intestine	fasted	AI11	HiSeq	NEB Next	2013	SE50	pRNA-B
Small intestine	fasted	U25	HiSeq	NEB Next	2013	SE50	pRNA-A
Small intestine	fasted	AI6_1	HiSeq	Illumina Truseq	2011	SE50	s1
Small intestine	fasted	AI6_2	GAIIx	Illumina Truseq	2011	PE120	SP03
Small intestine	fasted	AI11_1	HiSeq	Illumina Truseq	2011	SE50	s1
Small intestine	fasted	AI11_2	GAIIx	Illumina Truseq	2011	PE120	SP03
Small intestine	fasted	AJ6_1	HiSeq	Illumina Truseq	2011	SE50	s1
Small intestine	fasted	AJ6_2	GAIIx	Illumina Truseq	2011	PE120	TC05
Small intestine	fasted	AJ6_3	GAIIx	Illumina Truseq	2011	PE120	TC05
Small intestine	1DPF	Z12_1	HiSeq	Illumina Truseq	2011	SE50	s1
Small intestine	1DPF	Z12_2	GAIIx	Illumina Truseq	2011	PE120	SP03
Small intestine	1DPF	Z14_1	HiSeq	Illumina Truseq	2011	SE50	s1
Small intestine	1DPF	Z14_2	GAIIx	Illumina Truseq	2011	PE120	SP03
Small intestine	1DPF	Z14_3	GAIIx	Illumina Truseq	2011	PE120	TC05
Small intestine	1DPF	Z18_1	HiSeq	Illumina Truseq	2011	SE50	s1
Small intestine	1DPF	Z18_2	GAIIx	Illumina Truseq	2011	PE120	SP03
Small intestine	1DPF	V43	HiSeq	NEB Next	2013	SE50	pRNA-B
Small intestine	1DPF	Z18_3	HiSeq	NEB Next	2013	SE50	pRNA-B
Small intestine	4DPF	Y24	HiSeq	NEB Next	2013	SE50	pRNA-B
Small intestine	4DPF	Y5_1	HiSeq	Illumina Truseq	2011	SE50	s1
Small intestine	4DPF	Y5_2	GAIIx	Illumina Truseq	2011	PE120	SP03
Small intestine	4DPF	Y18_1	HiSeq	Illumina Truseq	2011	SE50	s1

Small intestine	4DPF	Y18_2	GAIIx	Illumina Truseq	2011	PE120	SP03
Small intestine	4DPF	Y18_3	GAIIx	Illumina Truseq	2011	PE120	TC05
Small intestine	4DPF	Y23_1	HiSeq	Illumina Truseq	2011	SE50	s1
Small intestine	4DPF	Y23_2	GAIIx	Illumina Truseq	2011	PE120	SP03

Table S2. The number of genes involved in each pathway as defined by IPA, the number of genes in the pathway that were assigned python orthologs via tblastx, and the number of those python orthologs observed with a non-zero level of expression in our dataset.

Pathway	Organ	Number of Genes	Number of genes assigned an orthologous python gene	Number of genes assigned an orthologous python gene and observed as expressed in dataset
mTOR	Heart	199	172	169
	Kidney			170
	Liver			167
	Small Int.			171
NRF2	Heart	292	223	220
	Kidney			222
	Liver			218
	Small Int.			220
